# Transcriptomics-Based Screening Identifies Pharmacological Inhibition of Hsp90 as a Means to Defer Aging

**DOI:** 10.1016/j.celrep.2019.03.044

**Published:** 2019-04-09

**Authors:** Georges E. Janssens, Xin-Xuan Lin, Lluís Millan-Ariño, Alan Kavšek, Ilke Sen, Renée I. Seinstra, Nicholas Stroustrup, Ellen A.A. Nollen, Christian G. Riedel

**Affiliations:** 1 Integrated Cardio Metabolic Centre (ICMC), Karolinska Institute, 14157 Huddinge, Sweden; 2 Department of Biosciences and Nutrition, Karolinska Institute, 14157 Huddinge, Sweden; 3 European Research Institute for the Biology of Ageing, University Medical Center Groningen, University of Groningen, 9700AD Groningen, the Netherlands; 4 Centre for Genomic Regulation (CRG), The Barcelona Institute of Science and Technology, 08003 Barcelona, Spain; 5 Universitat Pompeu Fabra (UPF), 08002 Barcelona, Spain

**Keywords:** aging, drug discovery, machine learning, geroprotectors, *Caenorhabditis elegans*, Hsp90, monorden, tanespimycin, lifespan, healthspan

## Abstract

Aging strongly influences human morbidity and mortality. Thus, aging-preventive compounds could greatly improve our health and lifespan. Here we screened for such compounds, known as geroprotectors, employing the power of transcriptomics to predict biological age. Using age-stratified human tissue transcriptomes and machine learning, we generated age classifiers and applied these to transcriptomic changes induced by 1,309 different compounds in human cells, ranking these compounds by their ability to induce a “youthful” transcriptional state. Testing the top candidates in *C. elegans*, we identified two Hsp90 inhibitors, monorden and tanespimycin, which extended the animals’ lifespan and improved their health. Hsp90 inhibition induces expression of heat shock proteins known to improve protein homeostasis. Consistently, monorden treatment improved the survival of *C. elegans* under proteotoxic stress, and its benefits depended on the cytosolic unfolded protein response-inducing transcription factor HSF-1. Taken together, our method represents an innovative geroprotector screening approach and was able to identify a class that acts by improving protein homeostasis.

## Highlights

• Transcriptome-based age classifiers can distinguish young versus old tissues
• Application of age classifiers to drug-induced transcriptomes finds geroprotectors
• Validation of geroprotectors in *C. elegans* highlights Hsp90 inhibitors
• Hsp90 inhibitors act through HSF-1 to improve health and extend lifespan

## Graphical Abstract

**Figure:**
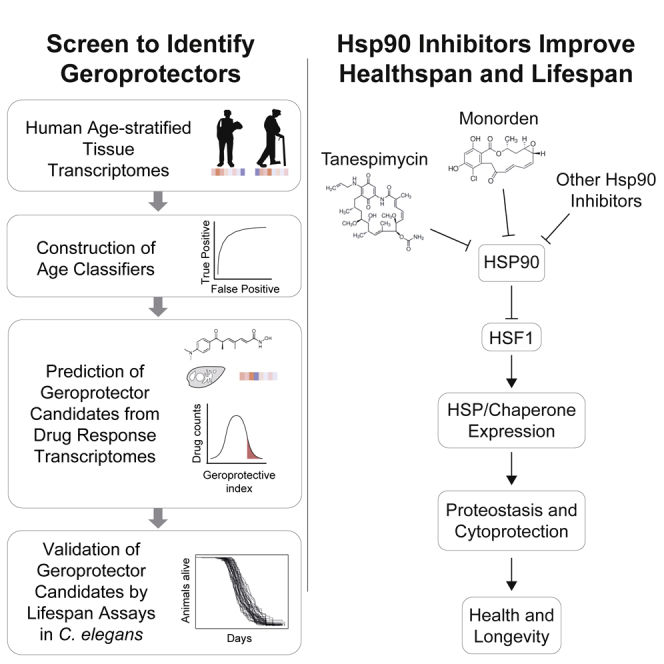

## Teaser

Identification of aging-preventive compounds in humans has been difficult. Here Janssens et al. combine age-stratified human tissue transcriptomes with drug response transcriptomes to identify compounds that lead to a “youthful” transcriptional state. By validation in *C. elegans*, the authors identify Hsp90 inhibitors that act through HSF-1 to promote health and longevity.

## Introduction

Aging is a major risk factor for many diseases and mortality (Niccoli and Partridge, 2012). Thus, targeting the aging process directly by pharmacological means could be a viable strategy to promote a healthier and longer life (Longo et al., 2015). Efforts are underway to explore these possibilities and to identify aging-preventive compounds, so-called “geroprotectors.” However, the list of candidates that are thought to confer such health and lifespan benefits to humans has remained very small (Barzilai et al., 2016, Kumar and Lombard, 2016). Even though screens covering tens of thousands of bio-active molecules have identified many drugs that extend the lifespan in simple model organisms (Lucanic et al., 2016, Petrascheck et al., 2007, Ye et al., 2014), validating their potential efficacy in humans is extremely time-consuming, limited in throughput, and restricted by ethical considerations. Thus, the sheer candidate numbers from such screens and their expected high frequency of a non-conserved effect in humans have discouraged their further evaluation (Kumar and Lombard, 2016).

So far, two major strategies have been tried to increase the probability of identifying compounds that are effective in humans. One approach has been to screen for them in mammalian laboratory models (e.g., mice, rats, or primates) but such studies are limited by significant costs, duration, and ethical considerations. The other approach has been to devise screening methodologies directly in humans that do not require treatment of individuals but limit themselves to compound screening in human cell culture models and the computational interpretation of the resulting data. This latter approach has been proven to be feasible, at least to identify dietary restriction mimetics (Calvert et al., 2016).

Here we try to take such cell culture- and computation-based approaches to a higher level of sophistication. Recent studies have demonstrated the power of human transcriptomes for biological age prediction (Peters et al., 2015, Sood et al., 2015, Yang et al., 2015). We make use of this predictive power by creating age classifiers from age-stratified human tissue transcriptomes (from the Genotype-Tissue Expression [GTEx] Consortium; GTEx Consortium, 2013, GTEx Consortium, 2015). We then use these classifiers to evaluate transcriptomic changes induced by 1,309 unique compounds in human cells (from the Connectivity Map [CMap]; Lamb et al., 2006) to identify geroprotectors. This results in numerous candidates, which we validate in the model organism *Caenorhabditis elegans.* Eventually, we focus on two of our top hits, the Hsp90 inhibitors monorden and tanespimycin. We show that they promote lifespan extension and better health, presumably through a mechanism that activates the transcription factor HSF1 and leads to improved protein homeostasis. Finally, we place our findings in the context of recent work that describes Hsp90 inhibitors as immuno-suppressants (Tukaj and Węgrzyn, 2016) and senolytics (Fuhrmann-Stroissnigg et al., 2017). Both of these functions are absent in *C. elegans* but should further potentiate the geroprotective benefits of Hsp90 inhibition when applied to humans.

## Results

### Construction of a Core Set of Transcriptome-Based Age Classifiers

**Figure 1:**
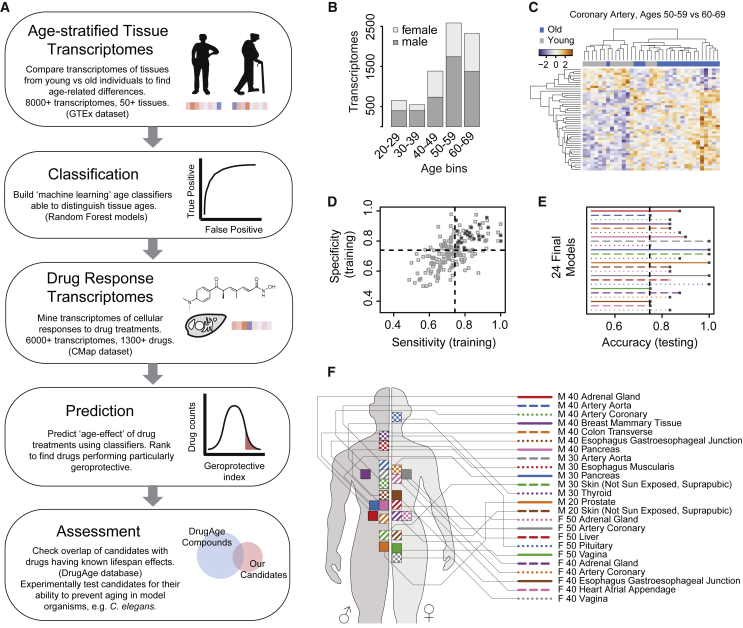
Study Strategy and Generation of Transcriptome-Based Age Classifiers (A) General strategy for the discovery of geroprotective drugs. Transcriptomes of young versus old individuals (step 1) are used to generate age classifiers (step 2). These classifiers are then applied to drug response transcriptomes (step 3) to identify compounds that change the transcriptome to a more youthful state. A ranking is generated, prioritizing compounds that are most likely to be geroprotective (step 4). Finally, we assess the highest-ranked compounds against known geroprotectors to estimate the efficacy of our prioritization method (step 5). (B) The age and gender demographics of the GTEx data used in this study. (C) Clustering of the differential gene expression data for a representative binary young (50–59) versus old (60–69) comparison performed in a particular tissue (coronary artery from females). Lines represent transcripts, and columns represent the individual tissue donors. Only transcripts used in the respective classification model are shown. (D and E) Inclusion criteria for the final classification models, demanding sensitivity (D), specificity (D), and accuracy (E) scores of above 0.75. (F) Distribution of the final 24 models among tissues, age bins, and genders. The letters M and F refer to the gender from which the model was generated (male and female, respectively). A number (20, 30, 40, or 50) denotes the young age decade from which the model was generated (either 20–29, 30–39, 40–49, or 50–59; these decades were always compared with the old decade of 60–69 years). The tissue names follow the GTEx naming scheme.

Recent studies have demonstrated that biological age can be estimated by machine learning approaches applied to healthy tissue transcriptome datasets (Peters et al., 2015, Sood et al., 2015). We reasoned that applying such biological age classifiers to drug-induced transcriptomes may reveal compounds with youth-inducing properties (Figure 1A). To test this, we turned to data available from the GTEx Consortium that contained a diverse set of human tissue transcriptomes originating from donors of various ages and both genders (GTEx Consortium, 2013, GTEx Consortium, 2015, Yang et al., 2015). GTEx transcriptomes were downloaded and preprocessed as described previously (GTEx Consortium, 2015, Melé et al., 2015, Taskesen and Reinders, 2016), resulting in a dataset of 8,555 transcriptomes from 51 tissues from both genders grouped into decade-sized age bins (ages 20–29, 30–39, 40–49, 50–59, and 60–69) (Figure 1B).

Next we took a binary classification approach to train machine learning models to distinguish “young” versus “old” ages. The transcriptomic data were separated by tissue and gender and kept in the decade-sized age bins. We defined being old as the decade of 60–69 years (the oldest decade in the GTEx data that matched our minimum sample number criteria) and, for each gender and tissue, made binary comparisons of this old age bin with any of the corresponding younger age bins. To minimize noise and limit the analysis to transcripts most likely to distinguish old from young samples, we filtered the datasets as follows. Of all possible binary comparisons, only those were made that contained at least 10 transcriptomes in each of the young and old datasets. The lowest 10% abundant transcripts were removed from the datasets. We further limited the analysis to genes that were differentially expressed between the two compared age bins. Finally, we filtered to only include genes present in the CMap data (Lamb et al., 2006). Figure 1C provides an example of a tissue taken from females (coronary artery) and pairwise age comparison (here, age bins 50–59 versus 60–69 years) that has undergone this processing, showing clustering of the resulting differential gene expression data. Using these filtered datasets, we generated random forest models and tuned them in an automated systematic manner (Kuhn et al., 2012, Liaw and Wiener, 2002; Figures 1 and S1), resulting in 182 age classification models of variable quality (Figure 1; Table S1). To generate a final core set of models, we then applied cutoff criteria based on model sensitivity, specificity, and accuracy from the training and testing phases (Figures 1D and 1E), ensuring that only the most effective classifiers remained. This resulted in 24 final models (Figure 1F; Table S2). These 24 models were comprised of 1,927 unique transcripts, most of which were unique to individual models (Table S2). Although the majority of these transcripts had no known age-related functions, several prominent aging-related genes were present in this list and contributed to the age classification, including glutathione S-transferase pi 1 (GSTP1; Ayyadevara et al., 2005, Mitsui et al., 2002, Umeda-Kameyama et al., 2007), insulin-like growth factor 1 receptor (IGF1R; Holzenberger et al., 2003, Kenyon et al., 1993, Tatar et al., 2001)), the sirtuin SIRT1 (Cohen, 2004, Herranz et al., 2010, Kaeberlein et al., 1999, Rogina and Helfand, 2004, Tissenbaum and Guarente, 2001), and mitochondrial uncoupling protein 2 (UCP2; Conti et al., 2006, Fridell et al., 2005)). A full list of the 1,927 genes and to which tissue models they contribute is provided in Table S2.

### Application of the Age Classifiers to Rank Compounds by Their Geroprotective Potential

Next we applied these 24 age classification models to detect geroprotective compounds (Figure 1A, steps 3–5). We turned to the publicly available CMap, a resource consisting of over 6,000 transcriptomes of various compound treatments performed on a selection of human cell lines (Lamb et al., 2006). In total, 1,309 different compounds are covered by this dataset, including Food and Drug Administration (FDA)-approved medications but also a variety of other bioactive molecules. CMap has successfully been used to find drugs affecting complex phenotypes; e.g., celastrol for the treatment of diabetes (Liu et al., 2015) and allantoin, which acts as a caloric restriction mimetic (Calvert et al., 2016).

**Figure 2:**
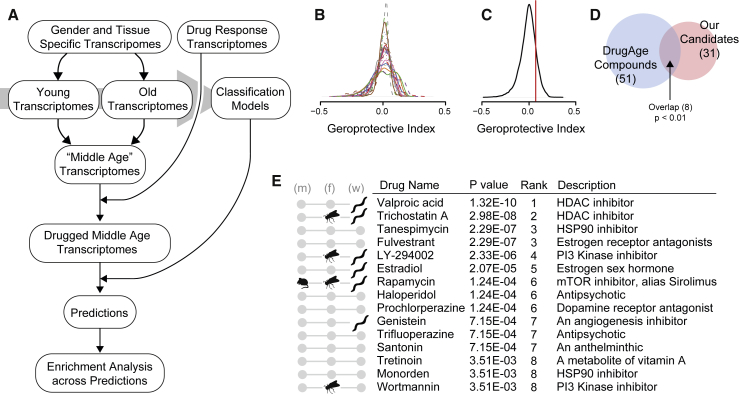
Drugs Ranked by Geroprotective Index (A) Pipeline for ranking CMap compounds by their geroprotective index. Tissues are separated into young and old groups used to build models. Middle age-representative transcriptomes are generated by averaging young and old. CMap drug response fold changes are applied to the middle age transcriptomes to generate “drug-induced” transcriptomes for each candidate drug. Age-classifying models predict the ages of the drug-induced transcriptomes. Enrichment scores are generated based on the 24 models’ separate predictions to find drugs most often ranked as geroprotective. (B) Geroprotective index ranking of CMap compounds for each of the 24 models (distribution of scores). The geroprotective index is a model’s prediction (the probability) of whether a transcriptome should be classified as young (see STAR Methods for details). (C) Consolidated geroprotective index scores (from B) of the CMap compounds (distribution, black line). The red line designates the mean absolute deviation, used as significance cutoff. (D) Overlap between our significant geroprotector candidates (31 total) and any CMap compounds that are listed as lifespan-extending in the DrugAge database (51 total) (p value generated by Fisher’s exact test). (E) The top 15 geroprotective candidate compounds resulting from our method. Compounds published to increase the lifespan in either mice (m), flies (f), or worms (w) are indicated.

We reasoned that a cell line’s exposure to aging-preventive compounds would induce transcriptional changes classified as young by our models (Figure 1A). Before we could apply our models to the CMap data, we first had to take into account that the CMap data and our GTEx-derived models originate from different types of cells with distinct baseline gene expression profiles. Thus, for each age classification model, we generated a prototypical “middle age” transcriptome comprised of each gene’s average expression level between the young and old age group of the GTEx data used to generate the model (STAR Methods). This resulted in transcriptomes that the models should not easily classify as either young or old (Figure 2A). Next we applied the fold gene expression changes of the drug responses in CMap to these prototypical middle age transcriptomes and asked our models whether this would shift the middle age transcriptome toward a young classification and, hence, a more youthful state. In this way, each CMap perturbation entry was systematically applied to all of the 24 models’ specific middle age transcriptomes (Figure 2A), resulting in 24 different predictions for each of CMap’s more than 6,000 entries (Table S2). Because many of the 1,309 compounds in the CMap often have been evaluated at various doses and incubation times or in different cell lines, and to give a drug the greatest possibility to show its geroprotective potential, we selected, for each drug, the CMap entry that gave the maximal probability of being young. This consolidated the dataset to 24 different predictions each for CMap’s 1,309 compounds. Furthermore, for better comparability between predictions from different models, we normalized model results to a maximal amplitude of 0.5, centered these probabilities around 0, and thereby created a “geroprotective index” in which a positive value signified prediction of a rejuvenated transcriptome (Figure 2B; Table S3).

To obtain a final evaluation for each drug, we assessed how often a drug was ranked as geroprotective by the 24 models. Although this approach may omit drugs with highly tissue-specific geroprotective effects, it prioritizes compounds that provide benefits across many tissues and, thus, might be particularly suitable for whole-organism treatment. We first consolidated the predictions from the 24 different models into one distribution and determined the mean absolute deviation (Figure 2C, red line) to establish a significance cutoff for our predictions. Then we counted how often a drug was predicted by one of the 24 models to have a geroprotective index above this cutoff and performed enrichment tests in relation to the whole distribution of predictions. After correcting for multiple-hypothesis testing, this resulted in a final significance-based ranking of drugs for their geroprotective potential (Table S3). We selected a corrected p value cutoff of 0.05 to form a candidate list of drugs for further consideration, which consisted of 31 compounds in total (Table S3; Figure 2E shows the top 15 compounds). To assess the efficacy of our method, we explored what was known about these 31 top candidates. Turning to DrugAge (Barardo et al., 2017), a database of compounds yielding lifespan effects in various model organisms, we found that, of its over 400 unique drugs that significantly increase the lifespan and, thus, are considered geroprotectors, 51 were present in CMap, and of these, 8 were in common with our list of 31 top candidates. This showed that our top candidates were significantly enriched for known geroprotective compounds (p < 0.01) (Figure 2D; Table S3), confirming that our screening method worked well.

Figure 2E shows the top 15 compounds from our candidate list, covering a wide range of mechanistic targets. These compounds include valproic acid and trichostatin A, two histone deacetylase (HDAC) inhibitors shown previously to increase the lifespan in worms (Calvert et al., 2016, Evason et al., 2008) and flies (Tao et al., 2004); the phosphatidylinositol 3-kinase (PI3K) inhibitors LY-294002 and wortmannin, shown to increase the lifespan in worms (Calvert et al., 2016) and flies (Danilov et al., 2013, Moskalev and Shaposhnikov, 2010); estradiol, shown to increase the lifespan in worms (Ye et al., 2014); the target of rapamycin (TOR) inhibitor rapamycin (also known as sirolimus), shown to extend the lifespan in worms (Calvert et al., 2016), flies (Bjedov et al., 2010), and mice (Harrison et al., 2009); and genistein, an angiogenesis inhibitor, shown to extend the lifespan in worms (Lee et al., 2015). Also several antidepressants were among our candidates, a class of drugs that has been shown previously to influence lifespan (Petrascheck et al., 2007, Zarse and Ristow, 2008). Finally, in ranks 16 to 31, we found several drugs that have been suggested to protect from all-cause mortality in epidemiological studies in humans: the diabetes therapeutic agent metformin (Bannister et al., 2014), the rheumatoid arthritis therapeutic agent methotrexate (Wasko et al., 2013), acetylsalicylic acid (aspirin) (Gum et al., 2001), as well as clozapine, a drug used to treat serious mental illness (Hayes et al., 2015).

Taken together, our screening approach was able to successfully rank compounds by their geroprotective potential, revealing a top candidate list significantly enriched for known lifespan-extending compounds. In addition, some previously undocumented geroprotector candidates were identified that should be interesting to investigate further.

For the scientific community, we provide an easy-to-use R script that is based on our classifiers and can be applied to make geroprotective predictions from any drug-induced transcriptomic changes in human cells (Figure S2; see STAR Methods for the download link).

### Validation of Geroprotector Candidates via *C. elegans*

To test for lifespan-extending capabilities among our candidate compounds, we turned to the nematode *C. elegans*, a relatively short-lived model organism frequently used in aging research, including drug screening for geroprotectors (Calvert et al., 2016, Carretero et al., 2015, Ye et al., 2014). We selected 29 compounds for evaluation. These included 14 of the top 15 compounds from our candidate list (p value cutoff, 3.6 × 10^−3^; Figure 2E); tanespimycin was initially omitted because of target redundancy with monorden and exceptional cost, but retested subsequently [see below]). Additionally, we included 15 compounds that we selected by hand (Table S3), irrespective of their ranking, either because we considered them promising geroprotector candidates based on existing annotations or to include drugs ranked poorly by our predictors (negative controls).

**Figure 3:**
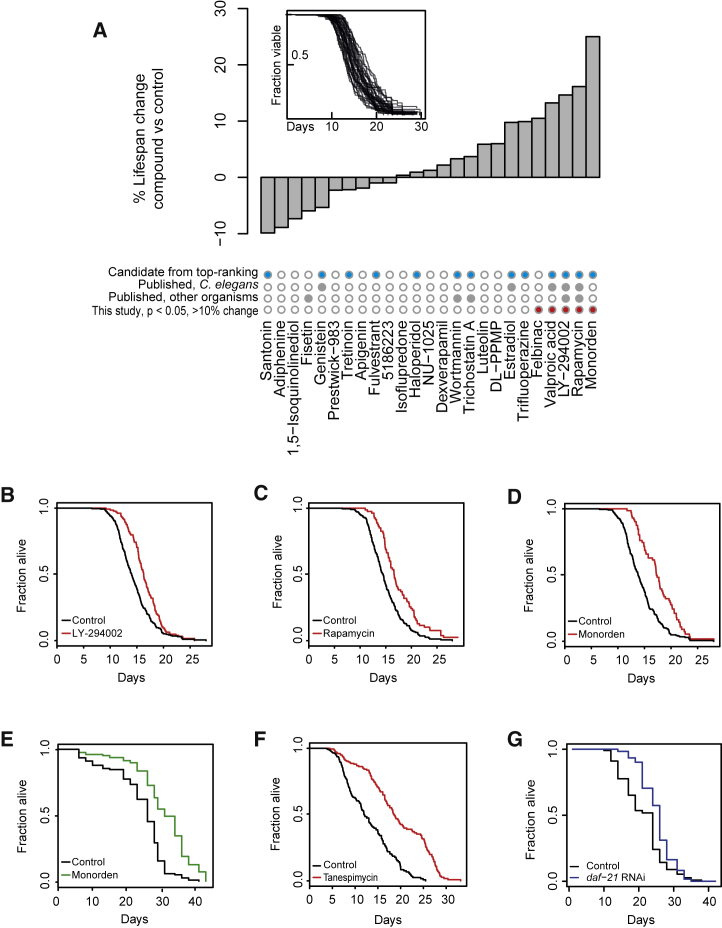
Lifespan Screening and Discovery of the Geroprotective Drugs Monorden and Tanespimycin (A) High-resolution lifespan curves were generated (inset), and changes in median lifespan were calculated for each candidate drug relative to the solvent control (bar graph). Compounds that were prioritized by our classification method (blue dots), that were already found to extend lifespan (gray dots), or that significantly extended lifespan in our study (p < 0.05 [log rank test] and >10% extension, red dots) are indicated. (B) The survival curve of LY-294002-treated worms from (A). (C) The survival curve of rapamycin (sirolimus)-treated worms from (A). (D) The survival curve of monorden-treated worms from (A). (E) The survival curve of monorden-treated worms, generated using the manual scoring method of prodding with a platinum wire. (F) The survival curve of tanespimycin-treated worms. (G) The survival curve of worms grown from the L4 stage on RNAi bacteria targeting *daf-21* (*daf-21* RNAi clone 1), the *C. elegans* gene encoding Hsp90. See Table S4 for drug concentrations, worm numbers and statistics, and Figures S3A–S3C for additional lifespan curves of compounds significantly increasing lifespan.

Compounds were evaluated at 50 μM (see Table S4 for solvents), a concentration that was deemed to be optimal in light of previously conducted *C. elegans*-based geroprotector screens (Calvert et al., 2016, Carretero et al., 2015, Ye et al., 2014). To obtain high-resolution lifespan data on this substantial panel of drugs, we used an automated imaging and analysis platform called the *C. elegans* “lifespan machine” (Stroustrup et al., 2013), which has repeatedly been proven to be a reliable tool (Lin et al., 2018, McEwan et al., 2016, Stroustrup et al., 2016). We applied the compounds to L4-stage larvae (animals near the end of their development), performed the survival assays, and eventually processed the data, making sure that each drug was tested using at least 50 worms. This resulted in a final list of 25 drugs giving high-quality lifespan data, displaying a range of lifespan phenotypes from an almost 10% decrease to more than a 25% increase (Figure 3A). Five compounds extended the lifespan significantly and by more than 10%: felbinac (an anti-inflammatory from a class of compounds known to extend worm lifespan; He et al., 2014), valproic acid, LY-294002, rapamycin, and monorden (Figure 3A, highlighted by red circles; Table S4; see Figures 3B–3D and Figures S3A and S3B for individual survival curves; we reproduced all these results by biological replicates). One compound (tyrphostin AG-1478, an epidermal growth factor receptor (EGFR) inhibitor; Fan et al., 1995) that significantly extended the lifespan but did not meet our inclusion criteria for worm numbers in the initial screen, was later validated with larger numbers of worms (Figure S3C; Table S4). Unfortunately, a few lifespan extension phenotypes reported previously by other labs could not be confirmed by our assays (i.e., genistein), but this may be due to different experimental setups and/or dosing.

Of the 25 tested compounds, the compounds prioritized by our classification models showed, in their mean, a reasonable lifespan extension (6%; Table S4), whereas such an effect was absent from the hand-selected compounds (−0.1%; Table S4). This is a remarkable outcome considering that we tested each compound only at a single dose and administered it only once (at the L4 stage), not optimizing treatment conditions and, thus, risking the occurrence of false negatives. Despite these limitations, we found a significant positive correlation between our ranking from the *in silico* analysis (Figure 2E; Table S3) and the compounds’ ability to extend lifespan in *C. elegans* (p = 0.02; Figure S3D; Table S5). Furthermore, the four compounds that exhibited the largest lifespan extension in our assays—monorden, rapamycin, LY-294002, and valproic acid (Figure 3A)—were all derived from our transcriptomics-based predictions, underscoring the power of our *in silico* approach to discover previously undocumented potent geroprotective compounds.

Turning back to the actual results of our assays, we conclude that we were able to confirm the well-known geroprotective effects of rapamycin and LY-294002, that we were able to support the lifespan-extending roles of valproic acid (which has recently been suggested to suffer from reproducibility issues; Lucanic et al., 2017) and tyrphostin AG-1478 (Ye et al., 2014), and that we discovered two previously undocumented geroprotective compounds: felbinac and monorden.

### Hsp90 Inhibition Extends the Lifespan of *C. elegans*

From here on, we decided to focus on monorden (also known as radicicol), which emerged as the most lifespan-extending candidate from our initial assays (Figure 3A; Table S4). Monorden is an established inhibitor of the chaperone protein Hsp90 (Griffin et al., 2004). Hsp90 helps to fold and, thus, assists with the function of many client proteins. Nevertheless, it also can sequester and inhibit proteins and their functions; e.g., the heat shock response transcription factor HSF1. Until now, monorden had remained unknown as a lifespan-extending drug. First, we validated its lifespan benefits by a conventional *C. elegans* lifespan assay using manual poking (Hamilton et al., 2005). Consistent with our lifespan machine data, we again observed a robust lifespan extension by approximately 25% at 50 μM (Figure 3E). We note that, in absolute numbers, the lifespans observed by the lifespan machine tended to be shorter than lifespans observed by manual scoring. Reasons could be different light exposure, different humidity, or slight shifts in temperature between the scoring methods. Nonetheless, monorden extended the lifespan in both setups, confirming the robustness of this finding. Because monorden is thought to target Hsp90, we next wanted to confirm that inhibition of this chaperone is indeed the mechanism by which the lifespan phenotypes are conferred. Coincidentally, we had identified tanespimycin (another Hsp90 inhibitor but structurally quite distinct from monorden; Blagosklonny, 2002, Schulte et al., 1998) as the third-best geroprotector candidate in our initial *in silico* screen (Figure 2E), although we had omitted it from initial validation in *C. elegans* because of target redundancy with monorden and the exceptional cost of the compound. Eventually testing the effects of tanespimycin at 25 μM, a lower dose to accommodate the cost of the drug, we likewise observed a substantial increase in lifespan (Figure 3F; Table S4).

Next we tested whether genetic perturbation of *daf-21,* the gene encoding Hsp90 in *C. elegans*, can lead to lifespan extension too. Prior work suggested that RNAi against *daf-21* starting from various developmental stages impairs growth or shortens the lifespan (Somogyvári et al., 2018). Nevertheless, when we knocked down *daf-21* from the L4 stage, we observed a significant lifespan extension, consistent with the effects observed with monorden or tanespimycin treatment (Figure 3G; Table S4). To better understand this potential discrepancy between our and prior findings, we determined the lifespan effects of *daf-21 RNAi*, comparing two different RNAi clones (one slightly weaker [1] and one stronger [2]; we consider clone 2 stronger because it can lead to developmental arrest, similar to a *daf-21* deletion [Birnby et al., 2000], whereas clone 1 does not), applying them from different stages of development or early adulthood and also testing the effect of diluting these RNAi clones and, thus, lowering their knockdown efficiency. Interestingly, we found that *daf-21* RNAi from early development (L1 or L2 stages) leads to either developmental arrest (when using RNAi clone 2 from L1) or, otherwise, lifespan shortening (Figure S4A), in line with prior work. Similarly, knockdown from day 1 of adulthood shortened the lifespan (Figure S4A). However, specifically *daf-21* RNAi from the L4 stage did not cause lifespan shorting. Instead, it extended the lifespan when using the weaker RNAi clone 1 (Figure S4A; Figure 3G). Next we tested the effect of diluting either of the *daf-21* RNAi clones in control RNAi and determined their lifespan effect when applied from the L4 stage. We found that lifespan extension phenotypes increased when we diluted the RNAi clones, with lifespan extension by the weaker RNAi clone 1 peaking at a concentration of 25% and the stronger RNAi clone 2 peaking at 10% (Figure S4B). These data argue that the timing of *daf-21* RNAi onset as well as the efficiency of the knockdown strongly affect the resulting lifespan phenotypes and that prior work may have missed a lifespan-extending phenotype from the L4 stage, likely because of stronger knockdown efficiency and/or slight differences in RNAi onset in their experiments.

Eventually, we conducted two more experiments to confirm that inhibitors like monorden or tanespimycin exert their phenotypes through Hsp90 inhibition. First, loss-of-function mutations in *daf-21* can promote Dauer formation (Birnby et al., 2000), a developmental arrest state of *C. elegans* that is exceptionally long-lived and resistant to environmental stress. Consistent with monorden inhibiting Hsp90, we found that it enhanced Dauer formation of wild-type animals at 27°C (Figure S4D; Table S4). Second, the lifespan benefits of monorden or tanespimycin should be suppressed by knockdown of *daf-21*. Testing this for monorden, we found that this was indeed the case (Figure S4C).

We conclude that Hsp90 inhibition, as long as it is mild and preferentially conducted from late development onward, extends the lifespan of *C. elegans* and that monorden or tanespimycin treatment from late development onward is a robust pharmacological way to confer it.

### Pharmacological Inhibition of Hsp90 Improves the Healthspan of *C. elegans*

**Figure 4:**
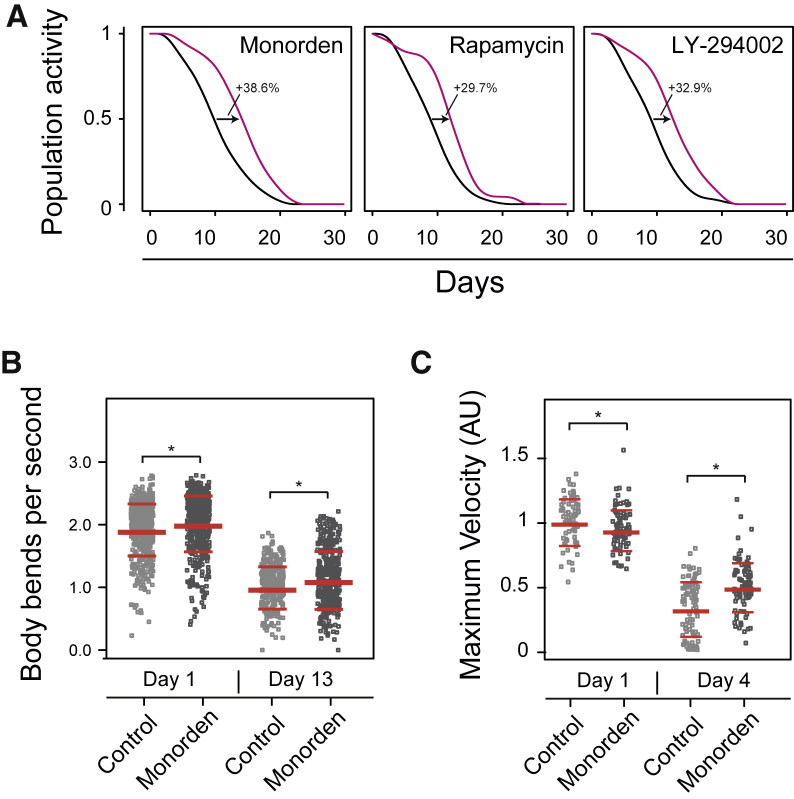
Health Benefits Resulting from Drug Treatments (A) The activity levels of different worm populations were assessed during aging (as described in Results and STAR Methods and further illustrated in Figure S5). Differences between activity curves are indicated. (B) Health as assessed by the animals’ thrashing frequency. Each dot represents a period of at least 2 s in a 1-min window during which at least 20 worms were followed. ^∗^p < 0.01 (Wilcoxon Mann Whitney test). Red lines indicate the median and quartiles. See Table S4 for worm numbers and statistics. (C) Health as assessed by the animals’ maximum velocity in early and mid-life (day 1 and day 4 of adulthood). Each dot represents one worm’s maximum velocity during a period of 30 s. ^∗^p < 0.01 (Wilcoxon Mann Whitney test). Red lines indicate the median and quartiles. See Table S4 for worm numbers and statistics.

Although living a longer life is tempting, even more importantly, we would like to increase the time in our life that we spend in good health, the so-called healthspan. To test for potential healthspan benefits from monorden treatment, we first turned again to the *C. elegans* lifespan machine. In addition to lifespan data, it also generates information regarding the position of worms throughout time. We reasoned that the degree of correlation of worm positions between successive time points provides information of the worm population’s activity level (Figure S5). Using this rather qualitative analysis, we found that monorden-treated worms had an extended period of active life, as did worms treated with the known geroprotectors rapamycin and LY-294002 (Figure 4A).

To validate this monorden-induced healthspan increase, we tested the motility of worms using a “thrashing” assay. This assay measures physical motility based on the swimming movements that *C. elegans* perform in liquid. We applied monorden to L4 worms and evaluated young (day 1 adulthood) and old (day 13 adulthood) animals. Comparing young and old worms, we saw a clear decline of the thrashing rate with age under control as well as treated conditions (Figure 4B). However, monorden treatment significantly increased the thrashing rate by up to 5% in young and, more importantly, by at least 12% in old worms (p < 0.05) (Figure 4B; Table S4).

A second powerful metric of *C. elegans* healthspan is their “maximum velocity.” It declines quite rapidly during adulthood, but when measured mid-life, it is predictive of the animals’ healthspan and lifespan (Hahm et al., 2015). Thus, we applied monorden to L4 animals and measured their maximum velocity during 30-s time frames on day 1 (young) and day 4 of adulthood (mid-life). We observed that monorden significantly improved animals’ maximum velocity in mid-life compared with controls (Figure 4C; Table S4).

Taken together, these findings show that monorden improves the healthspan in addition to conferring lifespan-extending effects, a phenotype extremely desirable when a geroprotective drug should be applied to humans for the benefit of healthy aging.

### Hsp90 Inhibitors Represent a Potential Pharmacological Class of Geroprotective Compounds that Act through the Heat Shock Transcription Factor HSF-1

**Figure 5:**
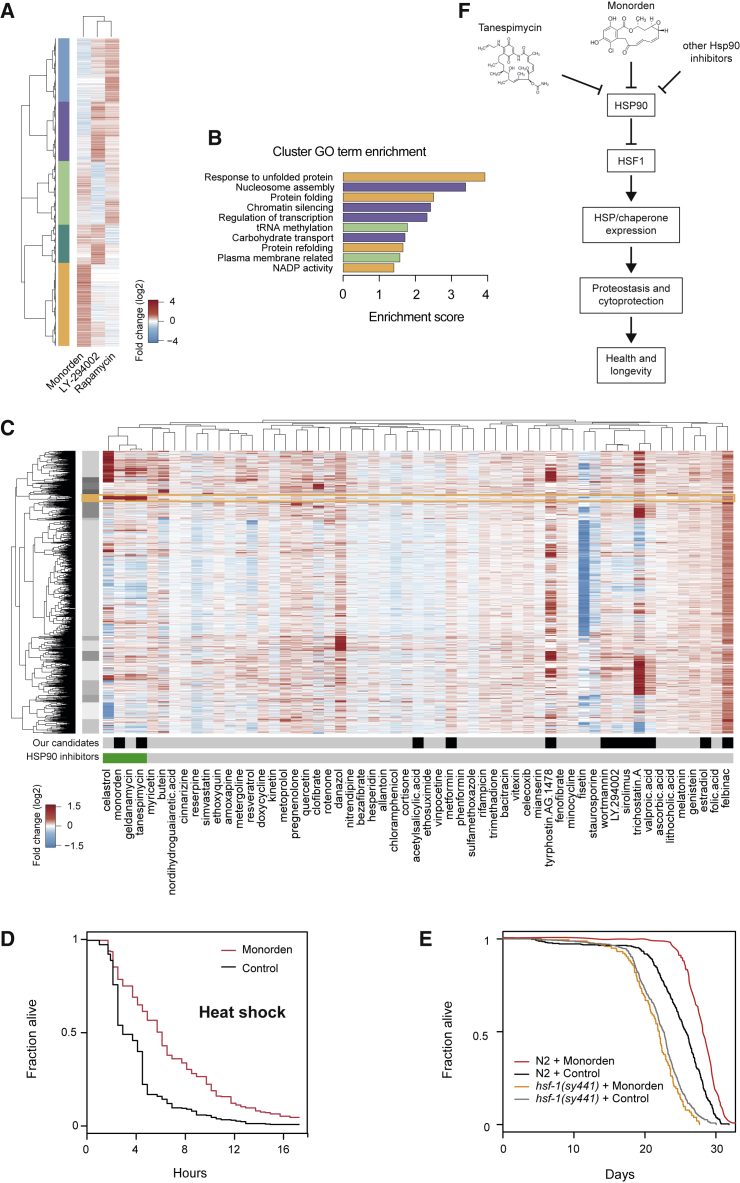
Hsp90 Inhibitors and the Transcriptional Landscape of Geroprotective Drugs (A) Heatmap clustering analysis of monorden-, LY-294002-, and rapamycin-induced transcriptomic changes (CMap data). Lines represent transcripts. Only those changing at least 1.5-fold are shown. Five main transcript clusters emerge, indicated in different colors: blue, purple, light green, dark green, and yellow. See Table S6 for the genes present in the transcript clusters. (B) The ten most enriched GO terms within the five clusters from (A). See Table S6 for all enrichments. (C) Heatmap clustering analysis of the transcriptomic changes induced by all CMap compounds that are listed as lifespan-extending in the DrugAge database, including previously undocumented compounds discovered by our study (monorden, tanespimycin, and felbinac). The bottom two rows indicate candidate compounds described in this study (black tiles) and any known Hsp90 inhibitors (green tiles). Lines represent transcripts; only transcripts changing at least 2-fold in any given drug were used for clustering and are shown. See Table S6 for all the transcripts used in the clustering analysis. The yellow box indicates a cluster of HSPs upregulated by monorden, tanespimycin, geldanamycin, and celastrol, all of which are known Hsp90 inhibitors. See Table S6 for the transcripts comprising and the GO term enrichments within the yellow cluster. (D) Heat shock survival assay performed at 35°C. See Table S4 for worm numbers and statistics. (E) Lifespan analysis for solvent control- and monorden-treated N2 and *hsf-1(sy441)* animals. See Table S4 for worm numbers and statistics. (F) Model of how Hsp90 inhibitors such as monorden and tanespimycin may confer their geroprotective effects. Hsp90 inhibitors lead to release of the transcription factor HSF1 from sequestration by Hsp90 proteins, allowing HSF1 to trimerize and activate transcription of its target genes in the nucleus, most importantly HSPs/chaperones. These HSPs/chaperones help to ensure the correct folding of proteins and prevent their aggregation with age, promoting protein homeostasis and cytoprotection, which lead to improved health and longevity.

To better understand how Hsp90 inhibition leads to these improvements in lifespan and healthspan, we investigated the transcriptional responses to monorden treatment, comparing them with those of the established geroprotectors rapamycin and LY-294002. From the original CMap transcriptomes, we chose three datasets generated from the same cell line (MCF7) as representative examples. Clustering analysis revealed that the effects of rapamycin and LY-294002 are much more similar to each other than they are to the effects of monorden (Figure 5A; Table S6). This may be expected because rapamycin and LY-294002 each target a single kinase in a nutrient-sensing pathway (Porta et al., 2014), whereas the effects of Hsp90 inhibitors should be much broader. In the same analysis, several transcript clusters emerged, defining the differences between the three treatments. One of these clusters stood out as being strongly upregulated upon monorden treatment but not upon treatment with rapamycin or LY-294002 (Figure 5A, yellow). Using Gene Ontology (GO) term enrichment analysis, we identified this cluster to be dominated by the unfolded protein response; i.e., an upregulation of heat shock proteins (HSPs) (Figure 5B; Table S6).

Having established the distinguishing ability of monorden to induce the unfolded protein response compared with rapamycin and LY-294002, we then asked how unique this ability really is, also in the broader landscape of all known geroprotectors. We thus extended our transcriptomic evaluation to all drugs that are described in DrugAge to extend lifespan and that are present in CMap. We generated transcriptomes representative of a drug response by averaging multiple instances of the same drug in CMap, and then we clustered these transcriptional profiles into a large heatmap, also including the geroprotective candidate compounds identified by our study (Figure 5C; Table S6). To see how the unfolded protein response was modulated in this transcriptional landscape and to determine whether this was a unique ability of monorden or Hsp90 inhibitors, we looked for transcript clusters particularly enriched for genes related to the unfolded protein response, and in particular the upregulation of HSPs and chaperones. One cluster in particular caught our attention (Figure 5C, highlighted in yellow; see Table S6 for GO enrichments in this cluster). This cluster is highly enriched for genes of the unfolded protein response, including HSPs from the HSP70 class (HSPA4L, HSPA6, and ASPA7), the HSP40 class (DNAJA1, DNAJC3, and DNAJB6), larger (HSPH1), and small (HSPB1) HSPs, among others (Table S6). Figure 5C shows that only four compounds are clear activators of this transcript cluster. Strikingly, all of these are known Hsp90 inhibitors: monorden, tanespimycin, celastrol, and geldenamycin. Celastrol is a pentacyclic triterpenoid (Hieronymus et al., 2006) described to induce heat shock response (Trott et al., 2008, Westerheide et al., 2004) and has been shown previously to increase the lifespan of *C. elegans* by 17% (Jung et al., 2014). Geldanamycin is a 1,4-benzoquinone ansamycin antibiotic. In *C. elegans*, a prior study failed to observe robust lifespan and healthspan benefits from this drug (Calvert et al., 2016). However, it has been argued that geldanamycin actually fails to bind *C. elegans* Hsp90 because of it harboring minor structural differences from the human protein (David et al., 2003). In light of the mostly side-effect-free lifespan benefits of the other three Hsp90 inhibitors, we therefore suggest that geldanamycin might indeed not function as an Hsp90 inhibitor in *C. elegans* and, instead, cause off-target effects. Taken together, the above observations show that Hsp90 inhibitors generally lead to an upregulation of HSPs/chaperones, a feature that is unique and defines them as a potential pharmacological class among geroprotectors.

It is well established that increased stress resistance can slow down the aging process and thereby improve organismal health and longevity. HSP upregulation confers such stress resistance by improving the organism’s protein homeostasis. Thus, we hypothesized that HSP upregulation comprises the mechanism by which Hsp90 inhibitors confer their beneficial effects on health and lifespan. To confirm this, we first tested whether a process that is particularly dependent on HSPs—namely, resistance to heat-induced unfolded protein stress—can be improved by monorden. Thus, we grew *C. elegans* at 20°C, exposed them to monorden from the L4 stage onward, and, on day 1 of adulthood, shifted them to 35°C. In line with our hypothesis, we found that monorden-treated animals survived significantly longer under these conditions (Figure 5D; Table S4). This observation shows that monorden not only extends the lifespan but also improves resistance to heat stress, an ability that is well known to depend on HSP induction (Wu, 1995). Further, it illustrates that upregulation of HSPs by monorden is functionally relevant and may well be the key mechanism of its geroprotective effects. To further support this notion, we turned to evaluation of the conserved transcription factor HSF-1. HSF-1 is a master regulator of the cytosolic unfolded protein response in eukaryotes, required for the upregulation of HSPs upon unfolded protein stress. HSF-1 protein abundance increases upon heat shock, and artificial overexpression of HSF-1 and the resulting HSP induction have been found to be sufficient to increase lifespan in worms (Hsu et al., 2003). In accordance with this, we observed a significant increase in HSF-1 protein levels upon monorden treatment (Figures S6A and S6B). Furthermore, we tested whether a hypomorphic allele of the *hsf-1* gene, *hsf-1(sy441)*, would be able to suppress the lifespan extension phenotype caused by monorden. Indeed, we found this to be the case (Figure 5E; Table S4).

Because HSF-1 is a transcription factor and its loss suppresses the lifespan benefits of monorden treatment, one would assume that monorden confers its benefits by HSF-1-induced transcriptional changes. To test this, we conducted gene expression analyses by mRNA sequencing (mRNA-seq). First, we treated wild-type *C. elegans* from the L4 stage onward with monorden or a solvent control and determined their transcriptomes on day 3 of adulthood to define the genes whose expression changes upon monorden treatment. This identified 825 transcripts that were induced and 993 transcripts that were repressed by monorden treatment (Figure S6C). Then we conducted the same comparison in *hsf-1(sy441)* mutant animals. Consistent with HSF-1 conferring much of the transcriptional changes induced by monorden treatment, most of the monorden-induced gene expression changes were lost in *hsf-1(sy441)* animals (Figure S6C). To further support the notion that monorden confers its effects through HSF-1, we determined the genes activated by HSF-1 upon heat shock and compared them with the genes activated by monorden in wild-type animals. Here we found a significant overlap between both gene sets (Figure S6D), showing that many of the genes HSF-1 activates under proteotoxic stress also become activated by monorden treatment.

In light of all these data, and because it has also been well established that Hsp90 functions as an inhibitor of HSF-1 (Zou et al., 1998), we eventually propose the following mechanistic cascade to cause prolonged lifespan and health in *C. elegans*: compounds like monorden and tanespimycin cause inhibition of Hsp90, which, in turn, causes activation of HSF-1. HSF-1, as a transcription factor, then upregulates the expression of heat stress response proteins, leading to improved protein homeostasis throughout age and, thereby, the compounds’ geroprotective effects (Figure 5F).

### Hsp90 Inhibition Shows Good Utility as a Combinatorial Treatment with Other Geroprotectors

**Figure 6:**
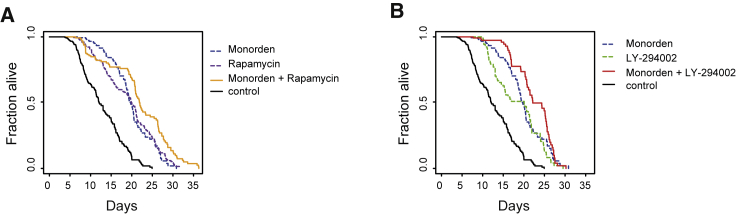
Additive Effects of Monorden Treatment with Other Geroprotective Compounds (A) Lifespan assay of worms treated with either a solvent control, monorden alone, rapamycin alone, or the combination of monorden and rapamycin. The latter combinatorial treatment shows an increased lifespan benefit (p < 0.01, log rank test). (B) Lifespan assay of worms treated with a solvent control, monorden alone, LY-294002 alone, or the combination of monorden and LY-294002; likewise, this shows additive benefits of the compounds (p < 0.01, log rank test). See Table S4 for worm numbers and statistics.

By now we had established that Hsp90 inhibitors lead to rather distinct transcriptional responses, including a unique ability to upregulate HSPs, compared with other geroprotectors in our transcriptome clustering analysis (Figure 5C). Thus, we hypothesized that Hsp90 inhibitors may provide excellent complementing capabilities when used in combinatorial treatments with other geroprotectors, resulting in additive beneficial effects on lifespan. To test this, we treated worms with 50 μM of monorden, rapamycin, or LY-294002 as well as combinations of 50 μM rapamycin and 50 μM Monorden or 50 μM LY-294002 and 50 μM monorden (Figure 6; Table S4). Indeed, monorden treatment was able to significantly extend the lifespan of either rapamycin-treated (p < 0.01) or LY-294002-treated (p < 0.01) animals by an additional ∼20%. In contrast, increasing the dose of monorden alone did not lead to additional lifespan extension (Figure S3E; Table S4). This latter finding is also consistent with monorden concentrations beyond 50 μM failing to further increase the monorden-driven induction of HSF-1 protein levels (Figure S6A).

In summary, monorden appears to target geroprotective pathways that are at least in part distinct from those targeted by rapamycin and LY-294002. This highlights Hsp90 inhibitors not only as good geroprotectors when used by themselves but also as compounds that may further enhance the benefits of other geroprotectors by targeting distinct but complementing pathways.

## Discussion

Here we established a powerful strategy for the discovery of geroprotective compounds using age classifiers derived from age-stratified human tissue transcriptomes and applying them to a database of drug-induced transcriptomic changes in human cell cultures. We reasoned that our strategy would be able to identify drugs that produce a “youthful” transcriptional signature and reveal candidate geroprotective compounds. Indeed, when applying this strategy to datasets from the GTEx consortium and CMap, we obtained geroprotector candidates, many of which we could validate as being lifespan-extending in *C. elegans*. This led to confirmation of the lifespan-extending abilities of known geroprotectors like tyrphostin AG-1478, LY-294002, and rapamycin. But more importantly, we identified geroprotectors that never before had been described to prevent aging or extend the lifespan in any organism; namely, felbinac, monorden, and tanespimycin. By eventually focusing on monorden and tanespimycin, we implied the existence of a very potent class of geroprotectors that act via Hsp90 inhibition and concomitant induction of stress response gene expression, in particular of the cytosolic unfolded protein response.

Hsp90 is a chaperone protein that accounts for about 1%–2% of all proteins in the cell (Powers and Workman, 2007, Welch and Feramisco, 1982, Welch et al., 1991) and that, together with co-chaperones and Hsp70, forms a sophisticated chaperone machinery (Pratt and Toft, 2003). Specifically, Hsp90 mostly serves to fold but sometimes also to regulate or even aid with the degradation of over 200 so-called “client proteins” that are enriched for both protein kinases and transcription factors (Pratt and Toft, 2003, Trepel et al., 2010). Several of the clients it helps to fold are growth-promoting or even oncogenic, including protein kinase B (AKT), mechanistic target of rapamycin (mTOR), ERBB2, BCR-ABL, C-RAF, CDK4, various steroid hormone receptors, and telomerase (Blagosklonny, 2002, Echeverría et al., 2011, Powers and Workman, 2007). Consistent with this growth-promoting role, substantial depletion of Hsp90 can lead to growth arrest during *C. elegans* development or tends to shorten the animals’ lifespan (Somogyvári et al., 2018; Figure S4A). Nevertheless, our work suggests that mild impairment of Hsp90 combined with the appropriate timing is actually beneficial, improving the organism’s healthspan and lifespan.

Because of its role in folding of growth-promoting kinases, Hsp90 has been widely acknowledged as a therapeutic target to fight cancer in humans (Calderwood et al., 2006, Powers and Workman, 2007, Whitesell and Lindquist, 2005). By now, pharma companies have filed over 30 patents for compounds that target Hsp90 (Sidera and Patsavoudi, 2014). Pharmacological impairment of Hsp90 can occur in a variety of ways. Most Hsp90 inhibitors block the ATP binding site of Hsp90 (e.g., monorden, tanespimycin, and geldanamycin; Prodromou et al., 1997, Schulte et al., 1998, Taipale et al., 2010), but others can also disrupt co-chaperone or client interactions (e.g., celastrol; Chadli et al., 2010) or interfere with post-translational modifications of Hsp90 (Li et al., 2009). The first described inhibitors of Hsp90 were naturally occurring compounds, including geldanamycin and monorden. Clinical exploration of geldanamycin was halted, however, because of toxic side effects. Eventually, structural analogs of geldanamycin with lower toxicity were developed (Blagosklonny, 2002, Supko et al., 1995). Tanespimycin is such an analog and has already led to more promising results in clinical trials (Banerji, 2009, Pacey et al., 2006, Sharp and Workman, 2006). Structural variants of monorden that improve its potency and stability have also been developed with promising results (Soga et al., 1999), and our work prompts their consideration as geroprotectors in the future.

Besides disrupting growth-promoting and oncogenic pathways, Hsp90 inhibitors also have a well-documented ability to induce the cytosolic unfolded protein response (Clarke et al., 2000, McCollum et al., 2006). This induction is thought to occur through the heat shock transcription factor HSF1, a client protein of Hsp90 (Trepel et al., 2010). Upon Hsp90 inhibition, HSF1 becomes activated by dissociating from Hsp90 and undergoing trimerization as well as nuclear translocation. When in the nucleus, HSF1 promotes the expression of heat shock response genes, including HSPs/chaperones (Zou et al., 1998). In the context of cancer therapy, this is an undesirable side effect of Hsp90 inhibitors because HSPs may serve to protect cancerous cells. To avoid these consequences, HSF1 repressors have been developed for combinatorial use with Hsp90 inhibitors (Trepel et al., 2010). In the context of aging, however, this activation of HSF1 should be beneficial. Consistently, overexpression of HSF1 has been shown to increase the lifespan in *C. elegans* (Baird et al., 2014, Hsu et al., 2003), and we show in Figure 5E that the lifespan-extending benefits of Hsp90 inhibition are abrogated by removal of HSF1 function. Taken together, we have identified Hsp90 inhibition as a potent strategy to improve healthspan and lifespan through the transcription factor HSF1 and, presumably, its ability to induce stress response genes; i.e., HSPs.

Interestingly, although Hsp90 inhibition leads, via this mechanism, to remarkable geroprotection in *C. elegans*, the geroprotective benefits in humans may even be greater. Previous work has shown that there are two hallmarks of human aging that do not exist in *C. elegans* and that additionally can be targeted by Hsp90 inhibition: (1) chronic inflammation and (2) the presence of senescent cells. Hsp90 inhibitors are able to suppress inflammation by blocking immune cell activation (Tukaj and Węgrzyn, 2016), and a recent screen identified Hsp90 inhibitors to be senolytic agents (Fuhrmann-Stroissnigg et al., 2017). Thus, Hsp90 inhibition might confer geroprotection through multiple mechanisms in humans, involving improved protein homeostasis (Conte et al., 2008, Griffin et al., 2004, Sonoda et al., 2010), as described in our study, but likely also through decreased inflammation (Zhao et al., 2013) and removal of senescent cells (Fuhrmann-Stroissnigg et al., 2017).

The findings of Fuhrmann-Stroissnigg et al. (2017) are of particular note because they observed that the Hsp90 inhibitor 17-DMAG could improve the healthspan in a progeroid mouse model, establishing Hsp90 inhibitors’ healthspan benefits in a mammalian context. In their study, Hsp90 inhibitor treatment correlated with an improved healthspan, which further correlated with a reduction in p16INK4a expression, a marker of senescent cells. Notably though, a causal link between clearance of senescent cells and the improved healthspan in these mice has yet to be shown, raising the possibility that the healthspan benefits observed in this study occurred independently of changes in senescent cells or may, at least in part, have been due to the mechanisms we propose in our work; namely, through HSF1 activation and improved protein homeostasis. Likewise, the authors demonstrated improvement of healthspan by Hsp90 inhibition in a progeroid model, but an improvement of healthspan and lifespan in a wild-type context still remains to be tested. Our work argues that both healthspan and lifespan will be improved in a wild-type animal context, too. In any case, the exciting fact that Hsp90 inhibitors influence multiple hallmarks of aging and the questions of how this occurs and whether these effects may synergize to improve health and lifespan in humans warrant further analysis.

Finally, we evaluated the utility of Hsp90 inhibitors for combinatorial treatments because they confer distinct and complementary gene expression changes compared with other known geroprotective drugs. Indeed, we could show *in vivo* in *C. elegans* that Hsp90 inhibition yields additive lifespan benefits when combined with mTOR or PI3K inhibitors. Thus, future studies of Hsp90 inhibitors as geroprotectors in humans should consider their combinatorial use, too.

Taken together, our study provides an innovative state-of-the-art approach to discover geroprotective compounds and highlights Hsp90 inhibition as a potential therapeutic approach to defer aging and age-related complications. Several well-developed Hsp90 inhibitors are already available that could be repurposed for such use and future work in humans will have to determine their full potential.

## STAR★Methods

### Key Resources Table

**Table:** 

REAGENT or RESOURCE	SOURCE	IDENTIFIER
**Antibodies**		

Rabbit polyclonal anti-HSF1	Thermo Fisher	Cat#PA3-017; RRID:AB_325968
Mouse monoclonal anti-Actin	Millipore	Cat#MAB1501; RRID:AB_2223041

**Bacterial and Virus Strains**		

*Escherichia coli* OP50-1	Caenorhabditis Genetics Center (CGC)	Cat# OP50-1; RRID:WB-STRAIN:OP50-1
*Escherichia coli* HT115, containing plasmid L4440	Kamath et al., 2003	N/A
*Escherichia coli* HT115, containing hsp90 RNAi plasmid #1	Reboul et al., 2003	N/A
*Escherichia coli* HT115, containing hsp90 RNAi plasmid #2	Morley and Morimoto, 2004	N/A

**Chemicals, Peptides, and Recombinant Proteins**		

Dimethyl sulphoxide	Sigma Aldrich	Cat#D2650
Valproic acid	Sigma Aldrich	Cat#S0930000
Trichostatin A	Sigma Aldrich	Cat#T8552
Tanespimycin	Sigma Aldrich	Cat#A8476
Fulvestrant	Sigma Aldrich	Cat#I4409
LY-294002	Sigma Aldrich	Cat#L9908
Estradiol	Sigma Aldrich	Cat#E8875
Rapamycin	Sigma Aldrich	Cat#37094
Haloperidol	Sigma Aldrich	Cat#H1512
Prochlorperazine	Sigma Aldrich	Cat#P9178
Genistein	Sigma Aldrich	Cat#G6649
Trifluoperazine	Sigma Aldrich	Cat#T8516
Santonin	Sigma Aldrich	Cat#Y0001052
Tretinoin	Sigma Aldrich	Cat#R2625
Monorden	Sigma Aldrich	Cat#R2146
Wortmannin	Sigma Aldrich	Cat#W1628
1,5-isoquinolinediol	Sigma Aldrich	Cat#I138
Dexverapamil	Sigma Aldrich	Cat#V4629
Fisetin	Sigma Aldrich	Cat#F4043
Luteolin	Sigma Aldrich	Cat#L9283
Cantharidin	Sigma Aldrich	Cat#C7632
DL-PPMP	Sigma Aldrich	Cat#P4194
Tyrphostin AG-1478	Sigma Aldrich	Cat#T4182
Apigenin	Sigma Aldrich	Cat#A3145
Adiphenine	Sigma Aldrich	Cat#A3649
Isoflupredone	Sigma Aldrich	Cat#1348907
Felbinac	Sigma Aldrich	Cat#Y0000731
Prestwick-983	Sigma Aldrich	Cat#D7571
NU-1025	Sigma Aldrich	Cat#N7287
5186223	ChemBridge	Cat#5186223

**Critical Commercial Assays**		

Direct-zol RNA MiniPrep kit	Zymo Research	Cat#R2052
TruSeq RNA SamplePrep V2 kit	Illumina	Cat#RS-122-2001/2

**Deposited Data**		

mRNA-seq data (deposited at the SRA (NCBI))	This study	PRJNA523315

**Experimental Models: Organisms/Strains**		

*C. elegans* N2 (wild type)	CGC	Cat# N2_(ancestral); RRID:WB-STRAIN:N2_(ancestral)
*C. elegans eri-1(mg366)*	CGC	Cat# GR1373; RRID:WB-STRAIN:GR1373
*C. elegans hsf-1(sy441)*	CGC	Cat# PS3551; RRID:WB-STRAIN:PS3551

**Software and Algorithms**		

OASIS 2	Han et al., 2016	https://sbi.postech.ac.kr/oasis2/
ImageJ	ImageJ	https://imagej.net/Welcome
GeroprotectorPredictor	This study	http://riedellab.org/downloads/GeroprotectorPredictor.7z
GraphPad Prism 7.05	GraphPad	https://www.graphpad.com/
Excel	Microsoft	https://products.office.com/en-us/?rtc=1
R	R Project	https://www.r-project.org/
TopHat 2	Trapnell et al., 2009	https://ccb.jhu.edu/software/tophat/index.shtml
Cuffdiff 2	Trapnell et al., 2010	http://cole-trapnell-lab.github.io/cufflinks/

### Contact for Reagent and Resource Sharing

Further information and requests for resources and reagents should be directed to and will be fulfilled by the Lead Contact, Christian G. Riedel (christian.riedel@ki.se).

### Experimental Model and Subject Details

*Caenorhabditis elegans* were maintained on NGM plates (Stiernagle, 2006) and fed with *Escherichia coli* OP50-1 unless stated otherwise. *C. elegans* strains used in this study were N2 (wild-type), GR1373 (*eri-1(mg366)*) (Kennedy et al., 2004), and PS3551 (*hsf-1(sy441)*) (Hajdu-Cronin et al., 2004), also listed in the Key Resources Table. All experiments were conducted using hermaphrodites. Animals were synchronized by bleaching and exposed to drugs or RNAi bacteria at different developmental or adult stages, which are indicated in the results, figure legends, or later sections.

### Method Details

#### Pre-processing of the GTEx data

The publicly available Version 6 of the GTEx Transcriptome datasets (accessed April 2016, dbGaP Accession phs000424.v6.p1) and annotation files (subject and sample attributes) were downloaded from the GTEx portal (https://gtexportal.org/home/datasets). Transcriptome data was loaded using the ‘read.gct’ function in the ‘CePa’ package (Centrality-based pathway enrichment) (Gu and Wang, 2013) into R. Data was preprocessed as previously described (GTEx Consortium, 2015, Melé et al., 2015, Taskesen and Reinders, 2016): Data was normalized to 1 million reads, low abundant transcripts were removed by selecting RPKMs whose values were less than 0.1 in at least 80% of samples. A pseudocount of 1 was added and data was log2-transformed. Transcriptomes were filtered to contain only those with an RNA integrity score (RIN) greater or equal to 6 (the SMRIN column of the annotation file) and for those that were deemed usable by the GTEx consortium (the SMAFRZE column of the annotation file). For data processing, GTEx files were parsed into sub files by tissue. In summary this resulted in a dataset of 8555 transcriptomes covering 19343 transcripts in 51 tissues. These sub files were divided further by gender and decade-sized age bins.

#### Processing of the GTEx data for age-related comparisons

Genders were treated separately, and all tissues were systematically processed in the same way. Tissues were individually loaded and parsed into age bins. ‘Old’ was considered to be the age bin of 60-69 (the age bin of 70-79 was omitted due to low sample numbers), and ‘young’ was considered to be any of the age bins of 20-29, 30-39, 40-49, or 50-59, which then would be compared to the old age-bin in a binary fashion. Young age groups were compared to the old, only when both groups contained at least 10 transcriptomes each. Next, the following steps were performed for the binary comparisons: Transcriptomes were reduced to only contain transcripts in common between the GTEx and CMap datasets (10,890 transcripts). Transcriptomes were further filtered to remove low abundant transcripts (the 10% least abundant). To aid the model building, a feature reduction step was undertaken by keeping only those transcripts which showed differential expression between the young and old age bin (p < 0.01). This was achieved by the ‘rowttests’ function in the R package ‘genefilter’ (Gentleman et al., 2017). Any cell line-based data in GTEx was omitted from our study.

#### Random Forest model generation and selection

For each binary comparison of ’old’ transcriptomes to any given ’young’ transcriptomes prepared above, data was reduced to have equal numbers of samples in each age group. Data was split into 70% used for model generation and 30% reserved for model validation, utilizing the ’createDataPartition’ function in the R package ‘caret’ (Kuhn et al., 2012). The 70% data partition was centered and scaled using the ’preProcess’ function of the same R package. Next, the R package ‘Random Forest’ (Liaw and Wiener, 2002) was implemented using the ‘train’ function again in the ‘caret’ package and was used to train models, using repeated cross validation (10-fold), using the default setting of 500 trees per forest, and using a standardized grid that varied the number of variables randomly selected per data split (varying .mtry in the tunegrid parameter). Models were automatically tuned, selecting the parameters with the best performing receiver-operating characteristic (ROC). Final models were tested on the remaining 30% data split for accuracy. 182 models were generated in this way. Performance of the models on the testing dataset was assessed using the ’performance’ function in the R package ‘ROCR’ (Sing et al., 2005). Final models were evaluated based on the ROC, the sensitivity, and the specificity achieved during model training, and the ROC and accuracy determined during model testing. Only those passing cutoff criteria above 0.75 in all these domains were retained. This resulted in 24 final models, our age-classifiers, able to distinguish young transcriptomes from those in the old age group of 60-69 years. Fourteen models derived from male tissues, from the adrenal gland (age 40-49), the coronary artery (age 40-49), mammary breast tissue (age 40-49), the transverse colon (age 40-49), the gastresophageal junction of the esophagus (age 40-49), the pancreas (age 40-49 and also 30-39), the aorta artery (30-39), the esophagus muscularis (age 30-39), suprapubic skin, not sun exposed (age 30-39 and also 20-29), the thyroid (age 30-39), and the prostate (age 20-29). Ten models derived from female tissues, from the adrenal gland (age 50-59, and also 40-49), the coronary artery (age 50-59, and also 40-49), the liver (age 50-59), the pituitary (age 50-59), the vagina (age 50-59, and also 40-49), the gastresophageal junction of the esophagus (age 40-49), and the heart’s atrial appendage (age 40-49).

#### Connectivity Map data preparation

Information on the Connectivity Map (CMap) instances (drug perturbation IDs and descriptions) was accessed through the R package ‘ConnectivityMap’ (Package and Shannon, 2013). A corresponding matrix of ’amplitudes’ (a measure of the extent of differential expression of a given probe set) was obtained by download from the Broad Institute’s FTP server (ftp://ftp.broadinstitute.org/distribution/cmap). The CMap amplitude matrix was converted to a fold change (log2) matrix using the conversion equation provided at https://www.broadinstitute.org/cmap/help_topics_frames.jsp. In order to later apply the CMap fold change data to the GTEx dataset, we converted the affymetrix probe IDs used in CMap to Ensemble gene IDs (used in GTEx) through the R package ‘biomaRt’ (Durinck et al., 2005, Durinck et al., 2009). For each GTEx gene entry, the corresponding probe fold change entries from CMap were collapsed to one value (if needed) in a conservative fashion by taking the median entry. Of the 19434 transcript entries in GTEx, we found 10869 to have corresponding CMap entries. Finally, the data was converted to linear scale, which resulted in a CMap dataset of fold changes that could be applied to transcriptomes from the GTEx dataset.

#### Generating ‘drug-induced’ transcriptomes for age classification

For each specific model, derived from a gender, tissue, and binary age group comparison within the GTEx dataset, the transcriptome of a corresponding ’prototypical’ age was generated, representing an average transcriptome between the compared old and young datasets. This was done by first determining the median transcriptional profile in the young and old datasets separately and subsequently averaging the two. This resulted in 24 prototypical transcriptomes, one for each model, which we termed ’middle age’. The processed CMap fold changes were then applied to each of these middle-aged transcriptomes, generating datasets that we term ’drug-induced’ transcriptomes, which are specific for each model. These transcriptomes are then ready to be used for age classification.

#### Application of age classifiers to ‘drug-induced’ transcriptomes and the eventual ranking of geroprotectors

“Drug-induced” transcriptomes were classified by their respective models, returning probability scores of being ‘young’. This resulted in 6100 CMap perturbation transcriptomes receiving a classification from each of the 24 models. For each drug present in replicate or various conditions in CMap, only a single score was used per model, namely the score from the most geroprotective prediction. This resulted in each drug being assessed for its geroprotective potential 24 times (once for each model), regardless of the number of replicates or conditions in CMap. To compare between models, these predictions were normalized to a maximal value of 0.5 and subsequently centered around 0, resulting in what we refer to as a ‘geroprotective index’. Finally, each drug was given a geroprotective ranked score, by seeing how many of the 24 predictions placed the drug above the mean absolute deviation of the consolidated distribution of all geroprotective index scores (Figure 2C), and a p value was generated using the hypergeometric function with a Benjamini correction.

#### DrugAge

The DrugAge database (Barardo et al., 2017) was accessed in May 2017, and was filtered to include all drugs that significantly increase lifespan.

#### Gene ID conversion

Where needed and unless otherwise specified, we used the BIOMART website (http://www.ensembl.org//useast.ensembl.org/biomart?redirectsrc=//www.ensembl.org%2Fbiomart) with the Human gene dataset (GRCh38.p10), to convert between Ensemble Stable Gene IDs and other gene names.

#### GO term enrichments

Gene functional enrichments were determined by using the DAVID Bioinformatics Resources (version 6.8) (Huang et al., 2009). Corresponding background gene lists were used for each enrichment analysis (see Table S6). Annotation clusters determined by DAVID (groupings of related genes based on the agreement of sharing similar annotation terms) having an enrichment score of > 1 were selected for consideration. A representative naming for the enrichment was selected after evaluation of the annotation cluster’s GO terms.

#### Data Visualizations

Data was visualized using R (R Development Core Team, 2013) with color schemes selected from there or the RColorBrewer package (Neuwirth, 2014).

#### RNAi by feeding

*C. elegans* were grown on the *E. coli* strain HT115 without plasmid until the required stage, then washed in M9 buffer containing antibiotics (Tetracyclin, Carbenicillin, and Streptomycin) to remove these bacteria, and finally transferred to HT115 containing dsRNA-expressing plasmids targeting *daf-21*. These *daf-21* RNAi bacteria were obtained from published sources: Clone 1 (Reboul et al., 2003); clone 2 (Morley and Morimoto, 2004). HT115 containing empty plasmid L4440 (Kamath et al., 2003) was used as a negative control.

#### Compounds

The following compounds were purchased from Sigma Aldrich: Dimethyl sulphoxide (DMSO, ref. D2650), Valproic acid (ref. S0930000), Trichostatin A (ref. T8552), Tanespimycin (ref. A8476), Fulvestrant (ref. I4409), LY-294002 (ref. L9908), Estradiol (ref. E8875), Rapamycin (ref. 37094), Haloperidol (ref. H1512), Prochlorperazine (ref. P9178), Genistein (ref.G6649), Trifluoperazine (ref. T8516), Santonin (ref. Y0001052), Tretinoin (ref. R2625), Monorden (ref. R2146), Wortmannin (ref. W1628), 1,5-isoquinolinediol (ref. I138), Dexverapamil (ref. V4629), Fisetin (ref. F4043), Luteolin (ref. L9283), Cantharidin (ref. C7632), DL-PPMP (ref. P4194), Tyrphostin AG-1478 (ref. T4182), Apigenin (ref. A3145), Adiphenine (ref. A3649), Isoflupredone (ref. 1348907), Felbinac (ref. Y0000731), Prestwick-983 (ref. D7571), NU-1025 (ref. N7287). The compound ‘5186223’ was purchased from ChemBridge. Compounds were dissolved in water, ethanol, or DMSO, in concentrations as specified (Table S4). For all compounds dissolved in DMSO, DMSO concentration in drug and control plates was kept at 0.33% (v/v), as recommended by several studies (Calvert et al., 2016, Ye et al., 2014). Ethanol concentrations were likewise kept at 0.33% (v/v). Two compounds, Fulvestrant and 5186223 required higher final DMSO concentrations (0.66% (v/v)) due to solubility issues.

#### Preparation of drugged NGM plates

NGM plates for lifespan and healthspan assays were identical to the maintenance plates mentioned above, with two exceptions: 1) Ampicillin (100 μg/ml) was added to the plates, and 2) the plates were seeded with dead instead of live OP50-1 bacteria, to avoid secondary effects from bacterial drug metabolism. The bacteria were killed by heat. Finally, drugs or solvent controls were added to the plates, and the plates then incubated over night at room temperature, before the worms were added.

#### Lifespan assays

*C. elegans* were grown in a synchronized way and divided among at least 4 plates (3 cm) per condition, with approximately 30 worms per plate. At the L4 stage, plates were treated with 0.1 g/ml 5-Fluoro-2′-deoxyuridine (FUDR) to prevent progeny production (Hamilton et al., 2005). The animals’ survival was recorded and analyzed by a fully automated ‘lifespan machine’, as previously described (Stroustrup et al., 2013). To ensure high quality results, lifespan curves were assessed, only if they were derived from at least 50 tracked worms. Data assessment was conducted using the ‘Survival’ package in R (Therneau, 2015) with Kaplan-Meier estimated survival curves compared to each other using the log-rank test. For the lifespan experiments shown in Figures 3E, 3G, and S4A, identical procedures were followed, except that survival of animals was examined manually every 2-3 days as previously described (Hamilton et al., 2005), and the data was assessed by OASIS2 (Han et al., 2016).

#### Heat-stress assay

Synchronized N2 animals were grown and transferred to drugged or solvent control NGM plates as described for lifespan assays. At day 1 of adulthood, animals were shifted to 35°C, and their survival was recorded and analyzed by the ‘lifespan machine’ as previously described (Stroustrup et al., 2013).

#### Healthspan analysis: Population activity assay

To derive ‘activity curves’ of worm populations as a healthspan measure from our ‘lifespan machine’ data, we utilized the ‘animal_position.csv’ output file that contains positions of ‘worm objects’ detected by the software. These are objects that the software presumes to be worms. We reconstructed the image space by creating a binary matrix corresponding to ‘non-worm’ (0) and ‘worm’ (1) positions, as indicated by the file, and as plotted in Figure S5A. Converting this binary image matrix to a linear vector allowed for Pearson’s correlations between time points to be calculated. We compared in this way every time point (n) with the one occurring directly subsequent to it (n+1) (see Figure S5A for illustration), generating a time-based correlation of worm positions (Figure S5B). A higher correlation between two adjacent time points was interpreted as less movement of the worms, indicative of lower ‘health’ of the population. Data from time points beyond the death of the worm population were omitted, to avoid artifacts. Combining these correlations from independent NGM plates, generated a large representative plot of time-based correlations, reflecting the worm population’s activity in time on the plates (Figure S5C). Fitting these correlations with a linear smoothing spline, using the preinstalled smooth.spline function in R, produced a curve that could be interpreted as movement of worms on the plates at the population level (Figure S5D). We subsequently made simple assumptions to plot this as a more familiar ‘curve’ for visual comparison between drug treatments and controls. Namely, 1) we mirrored and normalized the correlations, scaling them from 1 (‘most active’) to 0 (‘least active’), where we assumed that the highest correlation was the ‘least active’ time point and the lowest correlation was the ‘most active’ time point. 2) We assumed that the ‘most active’ time point represented the population optimum, and set all previous values to this. Further, we assumed that the ‘least active’ time point represented the end of movement on the plate, and set all subsequent time points to that value. Performing this for drug treatment and control conditions, allowed to assess a compound’s influence on the activity of a worm population as a measure of the population’s healthspan (Figure 4A).

#### Healthspan analysis: Maximum velocity assay

Individual worms’ maximum velocity was measured as previously described (Hahm et al., 2015). In brief, synchronized N2 animals were grown and transferred to drugged or solvent control NGM plates, as was done for lifespan assays. On day 1 and day 4 of adulthood, individual worms were transferred to individual NGM plates without bacteria. The worms’ movement was video-recorded immediately for 30 s (10 frames per second), and the velocity profile was analyzed using the ImageJ plugin ‘wrMTrck’ (Nussbaum-Krammer et al., 2015) and Microsoft Excel. Population distributions were assessed using the Wilcoxon-Mann-Whitney test from the preinstalled ‘wilcox.test’ function in R.

#### Healthspan analysis: Thrashing assay

Synchronized N2 animals were grown and transferred to drugged or solvent control NGM plates, as was done for lifespan assays. As the only difference, here 24-well plates were used. On day 1 and day 13 of adulthood, worms were measured for body bend movement in liquid. In brief, M9 was added to individual plate wells and videos were immediately recorded for a duration of 2 minutes (at 20 frames per second). Body bends were measured using the ImageJ plugin ‘wrMTrck’ (Nussbaum-Krammer et al., 2015). Population distributions were assessed using the Wilcoxon-Mann-Whitney test from the preinstalled ‘wilcox.test’ function in R.

#### Dauer assays

Wild-type *C. elegans* were synchronized by bleaching, the resulting eggs immediately dropped onto plates, and then grown for 52 hours at 27°C on life OP50-1 bacteria. Dauer formation was determined by scoring the animals’ survival after 30 min in 1% (w/v) SDS. Results were derived from four biological replicate experiments with > 100 worms per condition.

#### Whole worm lysis and western blotting

Around 200 animals were harvested, washed three times with cold M9 buffer, and then resuspended in cold M9 supplemented with Complete (Roche), 1 mM sodium orthovanadate, 25 mM sodium fluoride, and 20 mM β-glycerolphosphate. 5x Laemmli sample buffer was added to a final concentration of 1x and lysates were boiled for 6 minutes as well as ground for 15 s using a pestle homogenizer. The resulting lysates were subjected to SDS-PAGE and western blotting. Proteins were detected using anti-HSF1 antibody (Thermo Fisher, Cat# PA3-017, diluted 1:1000) or anti-Actin antibody (Millipore, Cat# MAB1501, diluted 1:1000) in TBST containing 5% (w/v) milk powder.

#### mRNA isolation, library construction, and high-throughput sequencing

Approximately 100 worms were collected by hand, washed with M9 buffer, and immediately frozen in liquid nitrogen. Total RNA was extracted using Trizol and a Direct-zol RNA MiniPrep kit (Zymo Research). mRNA-seq libraries were constructed using a TruSeq RNA SamplePrep V2 kit (Illumina). Multiplexed single-end sequencing of mRNA-seq libraries was conducted for 50 cycles on an Illumina HiSeq 3000 according to the manufacturer’s instructions. Image analysis, base calling and quality scoring were performed in real time with the standard Illumina analysis pipeline using a phiX control.

### Quantification and Statistical Analysis

For the automated lifespan and heat stress assays, survival data from the ‘lifespan machine’ was assessed using the ‘Survival’ package in R (Therneau, 2015). For manual lifespan assays, survival data was analyzed using OASIS2 (Han et al., 2016). In both cases, Kaplan-Meier estimated survival curves were compared to each other using the log-rank test. For the maximum velocity and thrashing healthspan assays, velocity and body bends were measured using the ImageJ plugin ‘wrMTrck’ (Nussbaum-Krammer et al., 2015) and Microsoft Excel, and population distributions were assessed using the Wilcoxon-Mann-Whitney test from the preinstalled ‘wilcox.test’ function in R. The exact number of animals used for each assay and further statistic details are indicated in the Supplementary Tables. For western blot quantification we used ImageJ. Statistical significance in western blot and dauer formation experiments was determined by two-tailed unpaired t test using GraphPad Prism 7.05. For all comparisons and assays, statistical significance was defined as p > 0.05.

#### Analysis of the mRNA-seq data

Reads were aligned to the *C. elegans* genome using the TopHat (v2.0.8b) software package (Trapnell et al., 2009), known gene model annotations (WS235), and the following parameters: --library-type fr-unstranded --b2-very-sensitive --min-coverage-intron 10 --min-segment-intron 10 --microexon-search --no-novel-juncs. Transcript abundance (FPKM, fragments per kilobase of transcript per million fragments) and differential expression were calculated using Cuffdiff (v2.1.1) included in the Cufflinks software package (Trapnell et al., 2010) using the following parameters: -u --FDR 0.05 --upper-quartile-norm --compatible-hits-norm --library-type fr-unstranded. All conditions were analyzed at least in biological duplicate. Replicates were individually mapped and then combined by Cuffdiff. All analyses were limited to protein-coding genes. Statistically significant differentially expressed genes (DEGs) were identified using a 5% FDR. Differential gene expression values were calculated as the ratio of FPKM values.

### Data and Software Availability

The high-throughput sequencing data generated by this study is available at the Sequence Read Archive at NCBI, under the accession number SRA: PRJNA523315. The R script to make geroprotective predictions can be downloaded at the following link: http://riedellab.org/downloads/GeroprotectorPredictor.7z

## Supplemental Information

Document S1. Figures S1–S6

Table S1. The 182 Generated Age Classification Models, related to Figure 1

Table S2. Information about the Final 24 Models, Related to Figures 1 and 2

Table S3. Geroprotective Potential of the CMap Drugs, Related to Figure 2

Table S4. Data and Statistics for the Lifespan, Healthspan, Heat Stress, and Dauer Assays, Related to Figures 3, 4, 5, 6, S3, and S4

Table S5. Correlation between Geroprotector Prediction and Lifespan Extension in *C. elegans*, Related to Figure 3

Table S6. Heatmap Data and GO Term Enrichments, Related to Figure 5

Document S2. Article plus Supplemental Information

## References

[bib1] Ayyadevara S., Engle M.R., Singh S.P., Dandapat A., Lichti C.F., Beneš H., Shmookler Reis R.J., Liebau E., Zimniak P. (2005). Lifespan and stress resistance of Caenorhabditis elegans are increased by expression of glutathione transferases capable of metabolizing the lipid peroxidation product 4-hydroxynonenal. Aging Cell.

[bib2] Baird N.A., Douglas P.M., Simic M.S., Grant A.R., Moresco J.J., Wolff S.C., Yates J.R., Manning G., Dillin A. (2014). HSF-1-mediated cytoskeletal integrity determines thermotolerance and life span. Science.

[bib3] Banerji U. (2009). Heat shock protein 90 as a drug target: some like it hot. Clin. Cancer Res..

[bib4] Bannister C.A., Holden S.E., Jenkins-Jones S., Morgan C.L., Halcox J.P., Schernthaner G., Mukherjee J., Currie C.J. (2014). Can people with type 2 diabetes live longer than those without? A comparison of mortality in people initiated with metformin or sulphonylurea monotherapy and matched, non-diabetic controls. Diabetes Obes. Metab..

[bib5] Barardo D., Thornton D., Thoppil H., Walsh M., Sharifi S., Ferreira S., Anžič A., Fernandes M., Monteiro P., Grum T. (2017). The DrugAge database of aging-related drugs. Aging Cell.

[bib6] Barzilai N., Crandall J.P., Kritchevsky S.B., Espeland M.A. (2016). Metformin as a Tool to Target Aging. Cell Metab..

[bib7] Birnby D.A., Link E.M., Vowels J.J., Tian H., Colacurcio P.L., Thomas J.H. (2000). A transmembrane guanylyl cyclase (DAF-11) and Hsp90 (DAF-21) regulate a common set of chemosensory behaviors in Caenorhabditis elegans. Genetics.

[bib8] Bjedov I., Toivonen J.M., Kerr F., Slack C., Jacobson J., Foley A., Partridge L. (2010). Mechanisms of life span extension by rapamycin in the fruit fly Drosophila melanogaster. Cell Metab..

[bib9] Blagosklonny M.V. (2002). Hsp-90-associated oncoproteins: multiple targets of geldanamycin and its analogs. Leukemia.

[bib10] Calderwood S.K., Khaleque M.A., Sawyer D.B., Ciocca D.R. (2006). Heat shock proteins in cancer: chaperones of tumorigenesis. Trends Biochem. Sci..

[bib11] Calvert S., Tacutu R., Sharifi S., Teixeira R., Ghosh P., de Magalhães J.P. (2016). A network pharmacology approach reveals new candidate caloric restriction mimetics in C. elegans. Aging Cell.

[bib12] Carretero M., Gomez-Amaro R.L., Petrascheck M. (2015). Pharmacological classes that extend lifespan of Caenorhabditis elegans. Front. Genet..

[bib13] Chadli A., Felts S.J., Wang Q., Sullivan W.P., Botuyan M.V., Fauq A., Ramirez-Alvarado M., Mer G. (2010). Celastrol inhibits Hsp90 chaperoning of steroid receptors by inducing fibrillization of the Co-chaperone p23. J. Biol. Chem..

[bib14] Clarke P.A., Hostein I., Banerji U., Stefano F.D., Maloney A., Walton M., Judson I., Workman P. (2000). Gene expression profiling of human colon cancer cells following inhibition of signal transduction by 17-allylamino-17-demethoxygeldanamycin, an inhibitor of the hsp90 molecular chaperone. Oncogene.

[bib15] Cohen H.Y. (2004). Calorie Restriction Promotes Mammalian Cell Survival by Inducing the SIRT1 Deacetylase. Science.

[bib16] Conte T.C., Franco D.V., Baptista I.L., Bueno C.R., Selistre-de-Araújo H.S., Brum P.C., Moriscot A.S., Miyabara E.H. (2008). Radicicol improves regeneration of skeletal muscle previously damaged by crotoxin in mice. Toxicon.

[bib17] Conti B., Sanchez-Alavez M., Winsky-Sommerer R., Morale M.C., Lucero J., Brownell S., Fabre V., Huitron-Resendiz S., Henriksen S., Zorrilla E.P. (2006). Transgenic mice with a reduced core body temperature have an increased life span. Science.

[bib18] Danilov A., Shaposhnikov M., Plyusnina E., Kogan V., Fedichev P., Moskalev A. (2013). Selective anticancer agents suppress aging in Drosophila. Oncotarget.

[bib19] David C.L., Smith H.E., Raynes D.A., Pulcini E.J., Whitesell L. (2003). Expression of a unique drug-resistant Hsp90 ortholog by the nematode Caenorhabditis elegans. Cell Stress Chaperones.

[bib20] Durinck S., Moreau Y., Kasprzyk A., Davis S., De Moor B., Brazma A., Huber W. (2005). BioMart and Bioconductor: a powerful link between biological databases and microarray data analysis. Bioinformatics.

[bib21] Durinck S., Spellman P.T., Birney E., Huber W. (2009). Mapping identifiers for the integration of genomic datasets with the R/Bioconductor package biomaRt. Nat. Protoc..

[bib22] Echeverría P.C., Bernthaler A., Dupuis P., Mayer B., Picard D. (2011). An interaction network predicted from public data as a discovery tool: application to the Hsp90 molecular chaperone machine. PLoS ONE.

[bib23] Evason K., Collins J.J., Huang C., Hughes S., Kornfeld K. (2008). Valproic acid extends Caenorhabditis elegans lifespan. Aging Cell.

[bib24] Fan Z., Lu Y., Wu X., DeBlasio A., Koff A., Mendelsohn J. (1995). Prolonged induction of p21Cip1/WAF1/CDK2/PCNA complex by epidermal growth factor receptor activation mediates ligand-induced A431 cell growth inhibition. J. Cell Biol..

[bib25] Fridell Y.W.C., Sánchez-Blanco A., Silvia B.A., Helfand S.L. (2005). Targeted expression of the human uncoupling protein 2 (hUCP2) to adult neurons extends life span in the fly. Cell Metab..

[bib26] Fuhrmann-Stroissnigg H., Ling Y.Y., Zhao J., McGowan S.J., Zhu Y., Brooks R.W., Grassi D., Gregg S.Q., Stripay J.L., Dorronsoro A. (2017). Identification of HSP90 inhibitors as a novel class of senolytics. Nat. Commun..

[bib27] Gentleman, A.R., Carey, V., Huber, W., and Hahne, F. (2017). Genefilter: methods for filtering genes from high-throughput experiments. R package version 1.58.0.

[bib28] Griffin T.M., Valdez T.V., Mestril R. (2004). Radicicol activates heat shock protein expression and cardioprotection in neonatal rat cardiomyocytes. Am. J. Physiol. Heart Circ. Physiol..

[bib29] GTEx Consortium (2013). The Genotype-Tissue Expression (GTEx) project. Nat. Genet..

[bib30] GTEx Consortium (2015). Human genomics. The Genotype-Tissue Expression (GTEx) pilot analysis: multitissue gene regulation in humans. Science.

[bib31] Gu Z., Wang J. (2013). CePa: an R package for finding significant pathways weighted by multiple network centralities. Bioinformatics.

[bib32] Gum P.A., Thamilarasan M., Watanabe J., Blackstone E.H., Lauer M.S. (2001). Aspirin use and all-cause mortality among patients being evaluated for known or suspected coronary artery disease: A propensity analysis. JAMA.

[bib33] Hahm J.-H., Kim S., DiLoreto R., Shi C., Lee S.-J.V., Murphy C.T., Nam H.G. (2015). C. elegans maximum velocity correlates with healthspan and is maintained in worms with an insulin receptor mutation. Nat. Commun..

[bib34] Hajdu-Cronin Y.M., Chen W.J., Sternberg P.W. (2004). The L-type cyclin CYL-1 and the heat-shock-factor HSF-1 are required for heat-shock-induced protein expression in Caenorhabditis elegans. Genetics.

[bib35] Hamilton B., Dong Y., Shindo M., Liu W., Odell I., Ruvkun G., Lee S.S. (2005). A systematic RNAi screen for longevity genes in *C. elegans*. Genes Dev..

[bib36] Han S.K., Lee D., Lee H., Kim D., Son H.G., Yang J.-S., Lee S.V., Kim S. (2016). OASIS 2: online application for survival analysis 2 with features for the analysis of maximal lifespan and healthspan in aging research. Oncotarget.

[bib37] Harrison D.E., Strong R., Sharp Z.D., Nelson J.F., Astle C.M., Flurkey K., Nadon N.L., Wilkinson J.E., Frenkel K., Carter C.S. (2009). Rapamycin fed late in life extends lifespan in genetically heterogeneous mice. Nature.

[bib38] Hayes R.D., Downs J., Chang C.K., Jackson R.G., Shetty H., Broadbent M., Hotopf M., Stewart R. (2015). The effect of clozapine on premature mortality: an assessment of clinical monitoring and other potential confounders. Schizophr. Bull..

[bib39] He C., Tsuchiyama S.K., Nguyen Q.T., Plyusnina E.N., Terrill S.R., Sahibzada S., Patel B., Faulkner A.R., Shaposhnikov M.V., Tian R. (2014). Enhanced longevity by ibuprofen, conserved in multiple species, occurs in yeast through inhibition of tryptophan import. PLoS Genet..

[bib40] Herranz D., Muñoz-Martin M., Cañamero M., Mulero F., Martinez-Pastor B., Fernandez-Capetillo O., Serrano M. (2010). Sirt1 improves healthy ageing and protects from metabolic syndrome-associated cancer. Nat. Commun..

[bib41] Hieronymus H., Lamb J., Ross K.N., Peng X.P., Clement C., Rodina A., Nieto M., Du J., Stegmaier K., Raj S.M. (2006). Gene expression signature-based chemical genomic prediction identifies a novel class of HSP90 pathway modulators. Cancer Cell.

[bib42] Holzenberger M., Dupont J., Ducos B., Leneuve P., Géloën A., Even P.C., Cervera P., Le Bouc Y. (2003). IGF-1 receptor regulates lifespan and resistance to oxidative stress in mice. Nature.

[bib43] Hsu A.-L., Murphy C.T., Kenyon C. (2003). Regulation of aging and age-related disease by DAF-16 and heat-shock factor. Science.

[bib44] Huang W., Sherman B.T., Lempicki R.A. (2009). Systematic and integrative analysis of large gene lists using DAVID bioinformatics resources. Nat. Protoc..

[bib45] Jung S.-K., Aleman-Meza B., Riepe C., Zhong W. (2014). QuantWorm: a comprehensive software package for Caenorhabditis elegans phenotypic assays. PLoS ONE.

[bib46] Kaeberlein M., McVey M., Guarente L. (1999). The SIR2/3/4 complex and SIR2 alone promote longevity in Saccharomyces cerevisiae by two different mechanisms. Genes Dev..

[bib47] Kamath R.S., Fraser A.G., Dong Y., Poulin G., Durbin R., Gotta M., Kanapin A., Le Bot N., Moreno S., Sohrmann M. (2003). Systematic functional analysis of the Caenorhabditis elegans genome using RNAi. Nature.

[bib48] Kennedy S., Wang D., Ruvkun G. (2004). A conserved siRNA-degrading RNase negatively regulates RNA interference in C. elegans. Nature.

[bib49] Kenyon C., Chang J., Gensch E., Rudner A., Tabtiang R. (1993). A C. elegans mutant that lives twice as long as wild type. Nature.

[bib50] Kuhn, M., Wing, J., Weston, S., Williams, A., Keefer, C., and Engelhardt, A. (2012). Caret: Classification and Regression Training. https://cran.r-project.org/web/packages/caret/index.html.

[bib51] Kumar S., Lombard D.B. (2016). Finding Ponce de Leon’s Pill: Challenges in Screening for Anti-Aging Molecules. F1000Res..

[bib52] Lamb J., Crawford E.D., Peck D., Modell J.W., Blat I.C., Wrobel M.J., Lerner J., Brunet J.-P., Subramanian A., Ross K.N. (2006). The Connectivity Map: using gene-expression signatures to connect small molecules, genes, and disease. Science.

[bib53] Lee E.B., Ahn D., Kim B.J., Lee S.Y., Seo H.W., Cha Y.S., Jeon H., Eun J.S., Cha D.S., Kim D.K. (2015). Genistein from vigna angularis extends lifespan in Caenorhabditis elegans. Biomol. Ther. (Seoul).

[bib54] Li Y., Zhang T., Schwartz S.J., Sun D. (2009). New developments in Hsp90 inhibitors as anti-cancer therapeutics: mechanisms, clinical perspective and more potential. Drug Resist. Updat..

[bib55] Liaw A., Wiener M. (2002). Classification and Regression by randomForest. R News.

[bib56] Lin X.-X., Sen I., Janssens G.E., Zhou X., Fonslow B.R., Edgar D., Stroustrup N., Swoboda P., Yates J.R., Ruvkun G., Riedel C.G. (2018). DAF-16/FOXO and HLH-30/TFEB function as combinatorial transcription factors to promote stress resistance and longevity. Nat. Commun..

[bib57] Liu J., Lee J., Salazar Hernandez M.A., Mazitschek R., Ozcan U. (2015). Treatment of obesity with celastrol. Cell.

[bib58] Longo V.D., Antebi A., Bartke A., Barzilai N., Brown-Borg H.M., Caruso C., Curiel T.J., de Cabo R., Franceschi C., Gems D. (2015). Interventions to slow aging in humans: Are we ready?. Aging Cell.

[bib59] Lucanic M., Garrett T., Yu I., Calahorro F., Asadi Shahmirzadi A., Miller A., Gill M.S., Hughes R.E., Holden-Dye L., Lithgow G.J. (2016). Chemical activation of a food deprivation signal extends lifespan. Aging Cell.

[bib60] Lucanic M., Plummer W.T., Chen E., Harke J., Foulger A.C., Onken B., Coleman-Hulbert A.L., Dumas K.J., Guo S., Johnson E. (2017). Impact of genetic background and experimental reproducibility on identifying chemical compounds with robust longevity effects. Nat. Commun..

[bib61] McCollum A.K., Teneyck C.J., Sauer B.M., Toft D.O., Erlichman C. (2006). Up-regulation of heat shock protein 27 induces resistance to 17-allylamino-demethoxygeldanamycin through a glutathione-mediated mechanism. Cancer Res..

[bib62] McEwan D.L., Feinbaum R.L., Stroustrup N., Haas W., Conery A.L., Anselmo A., Sadreyev R., Ausubel F.M. (2016). Tribbles ortholog NIPI-3 and bZIP transcription factor CEBP-1 regulate a Caenorhabditis elegans intestinal immune surveillance pathway. BMC Biol..

[bib63] Melé M., Ferreira P.G., Reverter F., DeLuca D.S., Monlong J., Sammeth M., Young T.R., Goldmann J.M., Pervouchine D.D., Sullivan T.J., GTEx Consortium (2015). Human genomics. The human transcriptome across tissues and individuals. Science.

[bib64] Mitsui A., Hamuro J., Nakamura H., Kondo N., Hirabayashi Y., Ishizaki-Koizumi S., Hirakawa T., Inoue T., Yodoi J. (2002). Overexpression of human thioredoxin in transgenic mice controls oxidative stress and life span. Antioxid. Redox Signal..

[bib65] Morley J.F., Morimoto R.I. (2004). Regulation of longevity in Caenorhabditis elegans by heat shock factor and molecular chaperones. Mol. Biol. Cell.

[bib66] Moskalev A.A., Shaposhnikov M.V. (2010). Pharmacological inhibition of phosphoinositide 3 and TOR kinases improves survival of Drosophila melanogaster. Rejuvenation Res..

[bib67] Neuwirth, E. (2014). RColorBrewer: ColorBrewer palettes. R Package version 1.1-2 https://cran.r-project.org/web/packages/RColorBrewer/index.html.

[bib68] Niccoli T., Partridge L. (2012). Ageing as a risk factor for disease. Curr. Biol..

[bib69] Nussbaum-Krammer C.I., Neto M.F., Brielmann R.M., Pedersen J.S., Morimoto R.I. (2015). Investigating the spreading and toxicity of prion-like proteins using the metazoan model organism C. elegans. J. Vis. Exp..

[bib70] Pacey S., Banerji U., Judson I., Workman P. (2006). Hsp90 inhibitors in the clinic. Handb. Exp. Pharmacol..

[bib71] Package, T., and Shannon, A.P. (2013). ConnectivityMap: Functional connections between drugs, genes and diseases as revealed by common gene-expression changes. https://www.bioconductor.org/packages/release/data/experiment/html/ConnectivityMap.html.

[bib72] Peters M.J., Joehanes R., Pilling L.C., Schurmann C., Conneely K.N., Powell J., Reinmaa E., Sutphin G.L., Zhernakova A., Schramm K., NABEC/UKBEC Consortium (2015). The transcriptional landscape of age in human peripheral blood. Nat. Commun..

[bib73] Petrascheck M., Ye X., Buck L.B. (2007). An antidepressant that extends lifespan in adult Caenorhabditis elegans. Nature.

[bib74] Porta C., Paglino C., Mosca A. (2014). Targeting PI3K/Akt/mTOR Signaling in Cancer. Front. Oncol..

[bib75] Powers M.V., Workman P. (2007). Inhibitors of the heat shock response: biology and pharmacology. FEBS Lett..

[bib76] Pratt W.B., Toft D.O. (2003). Regulation of signaling protein function and trafficking by the hsp90/hsp70-based chaperone machinery. Exp. Biol. Med. (Maywood).

[bib77] Prodromou C., Roe S.M., O’Brien R., Ladbury J.E., Piper P.W., Pearl L.H. (1997). Identification and structural characterization of the ATP/ADP-binding site in the Hsp90 molecular chaperone. Cell.

[bib78] R Development Core Team (2013). R: A language and environment for statistical computing.

[bib79] Reboul J., Vaglio P., Rual J.F., Lamesch P., Martinez M., Armstrong C.M., Li S., Jacotot L., Bertin N., Janky R. (2003). C. elegans ORFeome version 1.1: experimental verification of the genome annotation and resource for proteome-scale protein expression. Nat. Genet..

[bib80] Rogina B., Helfand S.L. (2004). Sir2 mediates longevity in the fly through a pathway related to calorie restriction. Proc. Natl. Acad. Sci. USA.

[bib81] Schulte T.W., Akinaga S., Soga S., Sullivan W., Stensgard B., Toft D., Neckers L.M. (1998). Antibiotic radicicol binds to the N-terminal domain of Hsp90 and shares important biologic activities with geldanamycin. Cell Stress Chaperones.

[bib82] Sharp S., Workman P. (2006). Inhibitors of the HSP90 molecular chaperone: current status. Adv. Cancer Res..

[bib83] Sidera K., Patsavoudi E. (2014). HSP90 inhibitors: current development and potential in cancer therapy. Recent Patents Anticancer Drug Discov..

[bib84] Sing T., Sander O., Beerenwinkel N., Lengauer T. (2005). ROCR: visualizing classifier performance in R. Bioinformatics.

[bib85] Soga S., Neckers L.M., Schulte T.W., Shiotsu Y., Akasaka K., Narumi H., Agatsuma T., Ikuina Y., Murakata C., Tamaoki T., Akinaga S. (1999). KF25706, a novel oxime derivative of radicicol, exhibits in vivo antitumor activity via selective depletion of Hsp90 binding signaling molecules. Cancer Res..

[bib86] Somogyvári M., Gecse E., Sőti C. (2018). DAF-21/Hsp90 is required for C. elegans longevity by ensuring DAF-16/FOXO isoform A function. Sci. Rep..

[bib87] Sonoda H., Prachasilchai W., Kondo H., Yokota-Ikeda N., Oshikawa S., Ito K., Ikeda M. (2010). The protective effect of radicicol against renal ischemia--reperfusion injury in mice. J. Pharmacol. Sci..

[bib88] Sood S., Gallagher I.J., Lunnon K., Rullman E., Keohane A., Crossland H., Phillips B.E., Cederholm T., Jensen T., van Loon L.J.C. (2015). A novel multi-tissue RNA diagnostic of healthy ageing relates to cognitive health status. Genome Biol..

[bib89] Stiernagle T. (2006). Maintenance of C. elegans. WormBook.

[bib90] Stroustrup N., Ulmschneider B.E., Nash Z.M., López-Moyado I.F., Apfeld J., Fontana W. (2013). The *Caenorhabditis elegans* Lifespan Machine. Nat. Methods.

[bib91] Stroustrup N., Anthony W.E., Nash Z.M., Gowda V., Gomez A., López-Moyado I.F., Apfeld J., Fontana W. (2016). The temporal scaling of Caenorhabditis elegans ageing. Nature.

[bib92] Supko J.G., Hickman R.L., Grever M.R., Malspeis L. (1995). Preclinical pharmacologic evaluation of geldanamycin as an antitumor agent. Cancer Chemother. Pharmacol..

[bib93] Taipale M., Jarosz D.F., Lindquist S. (2010). HSP90 at the hub of protein homeostasis: emerging mechanistic insights. Nat. Rev. Mol. Cell Biol..

[bib94] Tao D., Lu J., Sun H., Zhao Y.-M., Yuan Z.-G., Li X.-X., Huang B.-Q. (2004). Trichostatin A extends the lifespan of Drosophila melanogaster by elevating hsp22 expression. Acta Biochim. Biophys. Sin. (Shanghai).

[bib95] Taskesen E., Reinders M.J.T. (2016). 2D representation of transcriptomes by t-SNE exposes relatedness between human tissues. PLoS ONE.

[bib96] Tatar M., Kopelman A., Epstein D., Tu M.P., Yin C.M., Garofalo R.S. (2001). A mutant Drosophila insulin receptor homolog that extends life- span and impairs neuroendocrine function. Science.

[bib97] Therneau, T. (2015). A Package for Survival Analysis in S. https://cran.r-project.org/web/packages/survival/index.html.

[bib98] Tissenbaum H.A., Guarente L. (2001). Increased dosage of a sir-2 gene extends lifespan in Caenorhabditis elegans. Nature.

[bib99] Trapnell C., Pachter L., Salzberg S.L. (2009). TopHat: discovering splice junctions with RNA-Seq. Bioinformatics.

[bib100] Trapnell C., Williams B.A., Pertea G., Mortazavi A., Kwan G., van Baren M.J., Salzberg S.L., Wold B.J., Pachter L. (2010). Transcript assembly and quantification by RNA-Seq reveals unannotated transcripts and isoform switching during cell differentiation. Nat. Biotechnol..

[bib101] Trepel J., Mollapour M., Giaccone G., Neckers L. (2010). Targeting the dynamic HSP90 complex in cancer. Nat. Rev. Cancer.

[bib102] Trott A., West J.D., Klaić L., Westerheide S.D., Silverman R.B., Morimoto R.I., Morano K.A. (2008). Activation of heat shock and antioxidant responses by the natural product celastrol: transcriptional signatures of a thiol-targeted molecule. Mol. Biol. Cell.

[bib103] Tukaj S., Węgrzyn G. (2016). Anti-Hsp90 therapy in autoimmune and inflammatory diseases: a review of preclinical studies. Cell Stress Chaperones.

[bib104] Umeda-Kameyama Y., Tsuda M., Ohkura C., Matsuo T., Namba Y., Ohuchi Y., Aigaki T. (2007). Thioredoxin suppresses Parkin-associated endothelin receptor-like receptor-induced neurotoxicity and extends longevity in Drosophila. J. Biol. Chem..

[bib105] Wasko M.C.M., Dasgupta A., Hubert H., Fries J.F., Ward M.M. (2013). Propensity-adjusted association of methotrexate with overall survival in rheumatoid arthritis. Arthritis Rheum..

[bib106] Welch W.J., Feramisco J.R. (1982). Purification of the major mammalian heat shock proteins. J. Biol. Chem..

[bib107] Welch W.J., Kang H.S., Beckmann R.P., Mizzen L.A. (1991). Response of mammalian cells to metabolic stress; changes in cell physiology and structure/function of stress proteins. Curr. Top. Microbiol. Immunol..

[bib108] Westerheide S.D., Bosman J.D., Mbadugha B.N.A., Kawahara T.L.A., Matsumoto G., Kim S., Gu W., Devlin J.P., Silverman R.B., Morimoto R.I. (2004). Celastrols as inducers of the heat shock response and cytoprotection. J. Biol. Chem..

[bib109] Whitesell L., Lindquist S.L. (2005). HSP90 and the chaperoning of cancer. Nat. Rev. Cancer.

[bib110] Wu C. (1995). Heat shock transcription factors: structure and regulation. Annu. Rev. Cell Dev. Biol..

[bib111] Yang J., Huang T., Petralia F., Long Q., Zhang B., Argmann C., Zhao Y., Mobbs C.V., Schadt E.E., Zhu J., Tu Z., GTEx Consortium (2015). Synchronized age-related gene expression changes across multiple tissues in human and the link to complex diseases. Sci. Rep..

[bib112] Ye X., Linton J.M., Schork N.J., Buck L.B., Petrascheck M. (2014). A pharmacological network for lifespan extension in Caenorhabditis elegans. Aging Cell.

[bib113] Zarse K., Ristow M. (2008). Antidepressants of the serotonin-antagonist type increase body fat and decrease lifespan of adult Caenorhabditis elegans. PLoS ONE.

[bib114] Zhao Y., Huang Z.J., Rahman M., Luo Q., Thorlacius H. (2013). Radicicol, an Hsp90 inhibitor, inhibits intestinal inflammation and leakage in abdominal sepsis. J. Surg. Res..

[bib115] Zou J., Guo Y., Guettouche T., Smith D.F., Voellmy R. (1998). Repression of heat shock transcription factor HSF1 activation by HSP90 (HSP90 complex) that forms a stress-sensitive complex with HSF1. Cell.

