# Supplementary material for: Transcriptomics-Based Screening Identifies Pharmacological Inhibition of Hsp90 as a Means to Defer Aging
Source: Cell Rep. 2019 Apr 9;27(2):467–480.e6. doi: 10.1016/j.celrep.2019.03.044 (PMC6459000; doi:10.1016/j.celrep.2019.03.044)
Supplement: Document S1. Figures S1–S6 [file mmc1.pdf]

Cell Reports, Volume *27*

Supplemental Information

Transcriptomics-Based Screening Identifies Pharmacological Inhibition of Hsp90 as a Means to Defer Aging

Georges E. Janssens, Xin-Xuan Lin, Lluís Millan-Ariño, Alan Kavšek, Ilke Sen, Renée I. Seinstra, Nicholas Stroustrup, Ellen A.A. Nollen, and Christian G. Riedel

Janssens, et al., Figure S1

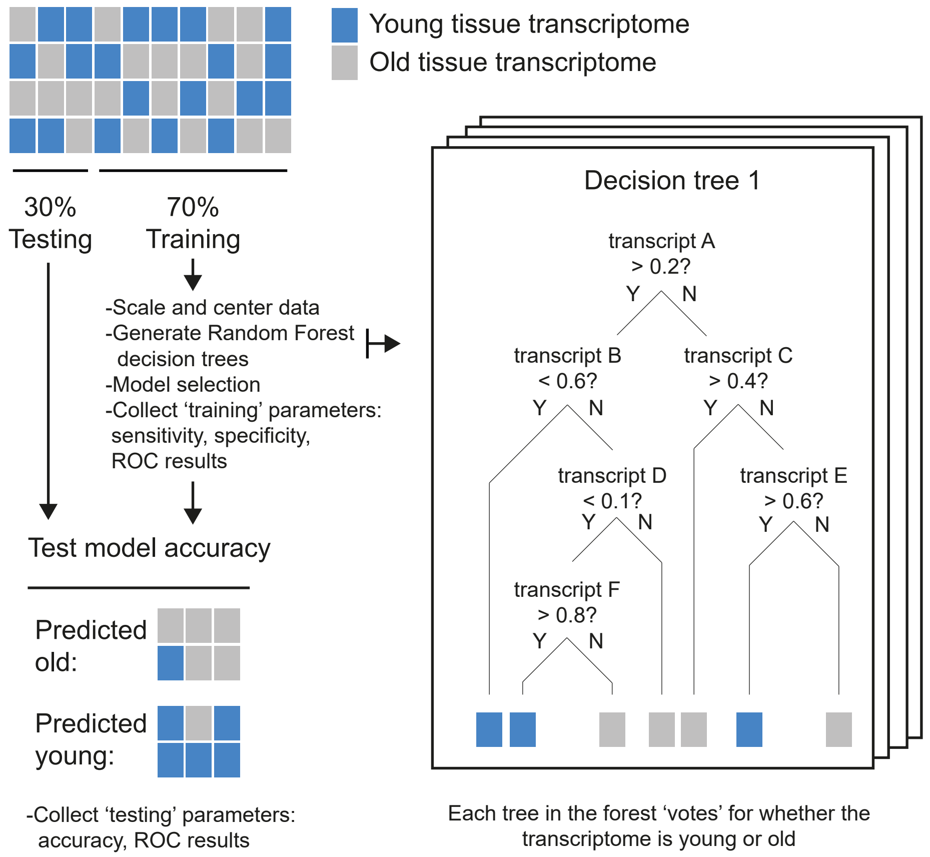

Figure S1. Generation of Age Classifying Models, related to Figure 1 and STAR Methods

Prior to age classification, the GTEx transcriptome datasets are separated by tissue, gender, and age into decade-wide age-bins, requiring at least 10 samples per bin. A particular ‘young’ and ‘old’ bin pair is selected, where the tissue and gender are identical, and where ‘old’ is defined as the 60-69 age bin, and ‘young’ is any of the younger age bins. Pairwise comparisons of transcripts between ‘young’ and ‘old’ are performed only on differentially expressed transcripts ($p<0.01$) to reduce the dataset and aid the classification. Data is subsequently downsampled to have equal numbers of old and young samples per comparison. 70% of the GTEx data is used to train models, by scaling and centering the data and eventually growing 500 random forest decision trees. Parameters of sensitivity, specificity, and ROC (receiver operating characteristic) are recorded in this ‘training’ phase. The models are tested against the remaining 30% of the GTEx data in the ‘testing’ phase to determine their accuracy and ROC in the independent data partition. Final models are selected, based on results from both, the training and testing phases.

Janssens, et al., Figure S2

A

Name
GeroprotectorPredictor_Script.R
GeroprotectorPredictor_WorkSpace.RData
predict_this_matrix.txt
▼ results
Final_graph.pdf
Final_table_raw.csv
Final_table_summary.csv

B

GeroprotectorPredictor_Script.R

```
################################################################################
#The script is organized as follows
#1) set work directory
#2) load required workspace
#3) load required libraries
#4) load the transcriptome(s) for geroprotective prediction(s)
      #predict_this_matrix.txt
      #ENSG gene names, fold change (NOT log transformed), can contain NAs
#5) prepare work space
#6) make age predictions
#7) convert to geroprotective index
#8) generate summary results
#9) visualize results
      #results/Final_graph.pdf
#10) generate final raw table
      #results/Final_table_raw.csv
#11) generate final summary table
      #results/Final_table_summary.csv
```

C

Final_table_raw.csv

	Percent_coverage	Monorden	Rapamycin	LY.294002
AdrenalGland	100	-0.0513698630136986	-0.123287671232877	-0.128424657534247
ArteryAorta	100	-0.201030927835052	-0.0360824742268041	-0.20446735395189
ArteryCoronary	100	0.228991596638655	0.138655462184874	0.191176470588235
BreastMammaryTissue	100	-0.150900900900901	0.351351351351351	-0.216216216216216
ColonTransverse	100	0.126923076923077	0.0403846153846155	0.253846153846154
EsophagusGastroesophagealJunction	100	0.111788617886179	0.0955284552845529	0.016260162601626
Pancreas	100	0.0535714285714286	0.173469387755102	0.178571428571429
ArteryAorta	100	-0.0709219858156028	0.00531914893617036	0.0354609929078015
EsophagusMuscularis	100	0.0654761904761906	0.176587301587302	0.0515873015873016
Pancreas	100	0.315126050420168	0.399159663865546	-0.0735294117647058
SkinNotSunExposedSuprapubic	100	0.0817843866171003	-0.0650557620817844	0.317843866171004
Thyroid	100	-0.0514705882352942	0.126838235294118	0.09375
Prostate	100	0.136200716845878	0.137992831541219	0.075268817204301
SkinNotSunExposedSuprapubic	100	-0.0809716599190283	-0.111336032388664	-0.0587044534412955
AdrenalGland	100	-0.072	-0.1	-0.088
ArteryCoronary	100	-0.0984251968503937	-0.118110236220472	-0.047244094488189
Liver	100	0.069811320754717	0.154716981132075	0.230188679245283
Pituitary	100	-0.149280575539568	-0.0611510791366907	-0.223021582733813
Vagina	100	-0.00362318840579712	-0.0181159420289855	0.0760869565217391
AdrenalGland	100	-0.0857142857142858	-0.0678571428571429	0.139285714285714
ArteryCoronary	100	0.136531365313653	-0.107011070110701	-0.0977859778597786
EsophagusGastroesophagealJunction	100	0.0859269282814614	0.0751014884979703	0.0277401894451962
HeartAtrialAppendage	100	0.0955882352941176	0.115808823529412	0.0477941176470588
Vagina	100	0.0829959514170041	0.0425101214574899	0.119433198380567

D

Final_graph.pdf

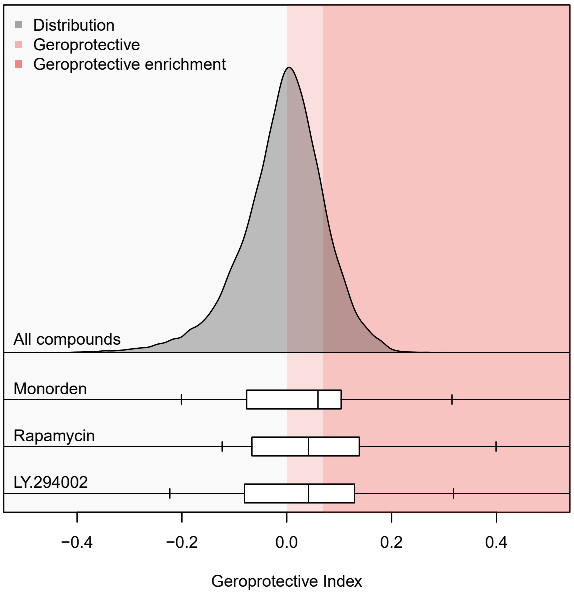

E

Final_table_summary.csv

	Monorden	Rapamycin	LY.294002
n.models	24	24	24
n.above.0	13	14	15
percent.above.0	54.167	58.333	62.5
n.above.MAD.MED	8	10	8
geroprotective.p.value.MED	0.04	0.004	0.04
geroprotective.p.value.corr.MED	0.278	0.081	0.278
significant.result.MED	No	No	No
n.above.MAD.MAX	11	11	10
geroprotective.p.value.MAX	0	0	0.001
geroprotective.p.value.corr.MAX	0.009	0.009	0.033
significant.result.MAX	Yes	Yes	Yes

Figure S2. Pipeline for the transcriptomics-based identification of geroprotector candidates, related to Figure 2 and STAR Methods

Accompanying this manuscript, a 'stand-alone' script is provided, which can be used to determine the geroprotective index scores of unknown compounds, based on their transcriptomic effects. The script is provided as a compressed file (GeroprotectorPredictor.7z) and available at http://riedellab.org/downloads/GeroprotectorPredictor.7z. Unpackaging it provides (A) an R script, all required data (models, variables, etc), and a modifiable txt file that can be used to input transcriptome(s). (B) An outline of the steps in the R script is provided. Running the R script produces results that are stored in a results folder. These include (C) the raw predictions from each model as geroprotective index scores, (D) a final graph showing the predictions of the input transcriptomes relative to all other predictions from CMap, and (E) a summary table providing final results, most importantly enrichment scores and their significance. Any greyed-out values were not used in this study but are explained in the instructions included with the R script.

Janssens, et al., Figure S3

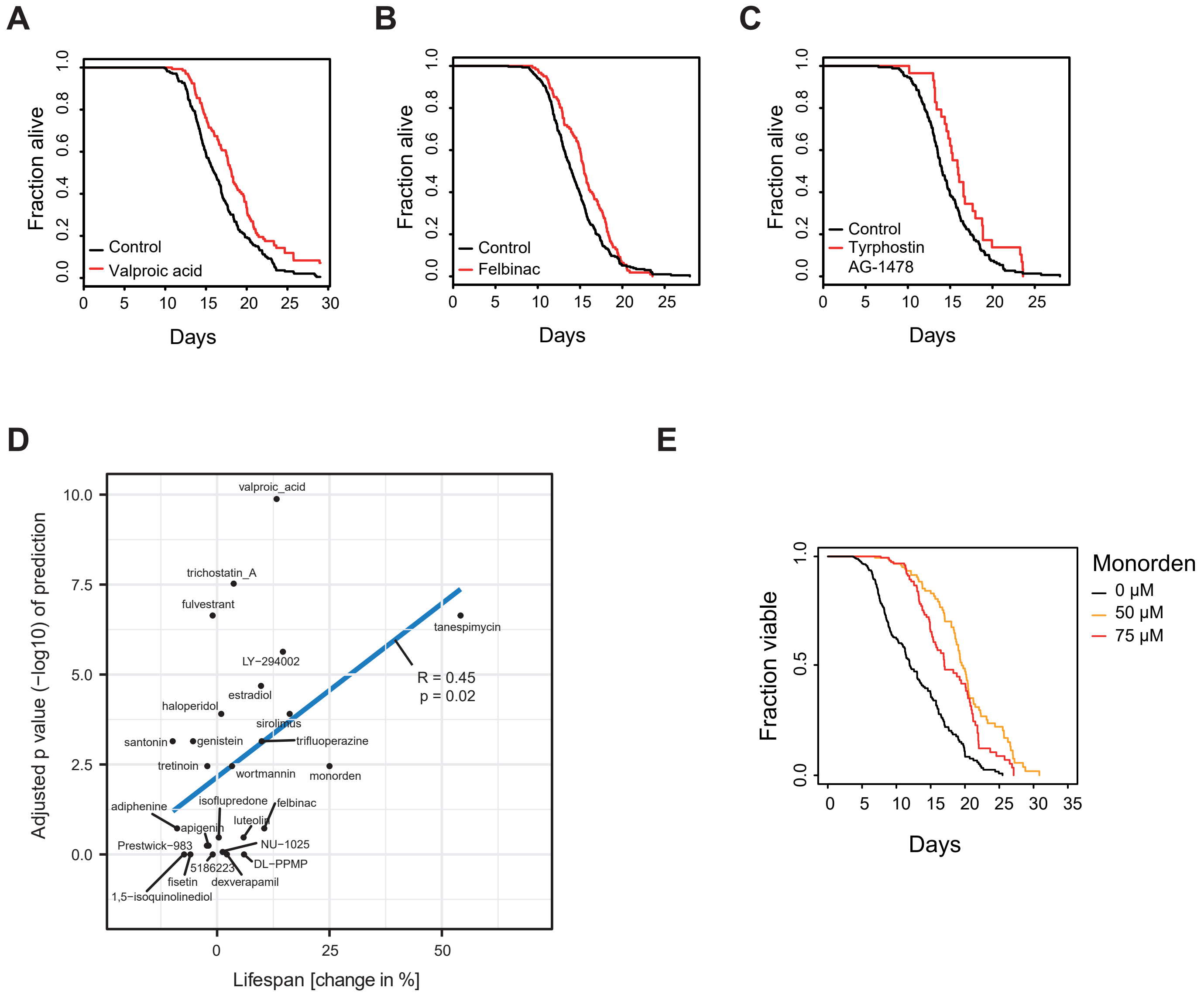

Figure S3. Lifespan curves of additional candidate compounds, the correlation between our geroprotector predictions and the compounds' impact on lifespan, and the dose response of Monorden treatment in *C. elegans,* related to Figures 3 and 6

(A-C) *C. elegans* survival curves of additional candidate compounds, showing a greater than 10% lifespan extension with a significance of $p < 0.05$. (A) The survival curve of Valproic acid treated worms. (B) The survival curve of Felbinac treated worms. (C) The survival curve of Tyrphostin AG-1478 treated worms. See Table S4 for drug concentrations, worm numbers and statistics. Tyrphostin AG-1478 results were omitted from Figure 3A due to insufficient worms in the first lifespan experiment (<50), though results were later confirmed with higher worm numbers (see Table S4). (D) For the compounds studied in Figure 3, their predicted geroprotective potentials correlate with their ability to extend lifespan in *C. elegans*. The -log10 adjusted p-values of their predictions were plotted against their lifespan phenotypes. The blue line describes a linear regression ($R = 0.45$, $p = 0.02$). (E) Survival curves of *C. elegans* treated either with 50 µM Monorden, 75 µM Monorden, or solvent control, showing that the lifespan benefits of Monorden may already have been maximal at a dose of 50 µM. See Table S4 for worm numbers and statistics.

Janssens, et al., Figure S4

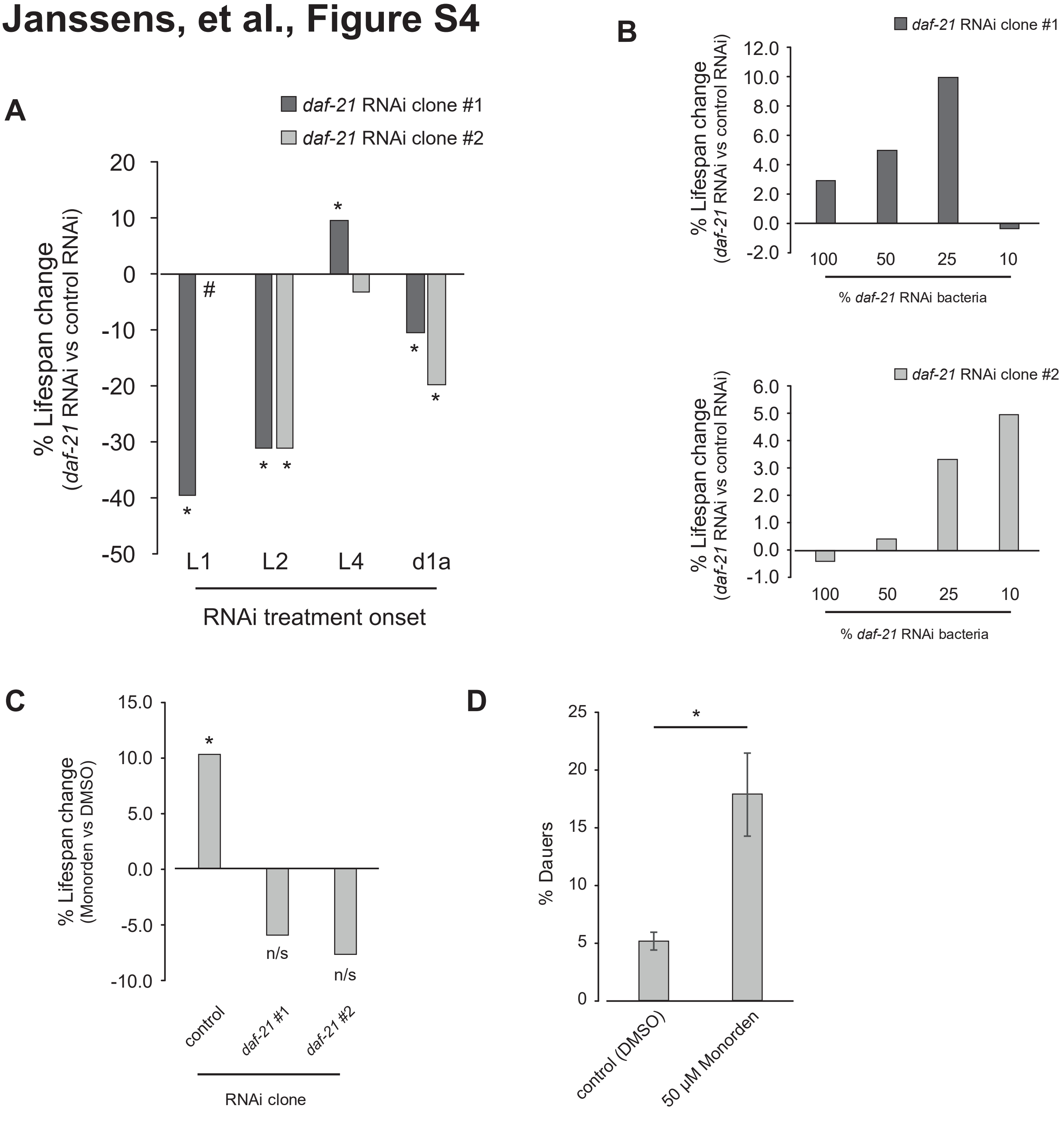

Figure S4. Impact of timing and extent of Hsp90 loss on lifespan, impact of Hsp90 loss on Monorden-induced lifespan benefits, and impact of Monorden treatment on Dauer formation in *C. elegans* , each related to Figure 3

(A,B) *daf-21* RNAi beginning from different times during development or conducted at different levels of efficiency leads to different lifespan phenotypes. (A) *C. elegans* were grown either from the L1 stage, L2 stage, L4 stage, or day 1 of adulthood on control or the indicated RNAi bacteria and their lifespan was monitored by manual scoring. Changes in median lifespan of *daf-21* RNAi treated animals versus control RNAi treated animals are shown. Lifespan assays were performed using *eri-1(mg366)* mutant animals, to yield better knockdown efficiency (Kennedy et al., 2004). Significant lifespan changes ($p < 0.05$ (log-rank test)) are indicated (*). Animals treated with *daf-21* RNAi clone 2 from the L1 stage arrested developmentally (#). See Table S4 for worm numbers and statistics. (B) *C. elegans* were grown from the L4 stage on either control RNAi bacteria or on *daf-21* RNAi bacteria diluted in control RNAi bacteria to the indicated concentrations. Survival was monitored by use of an automated 'lifespan machine'. Changes in median lifespan of *daf-21* RNAi treated animals versus control RNAi treated animals are shown. Lifespan assays were performed using *eri-1(mg366)* mutant animals, to yield better knockdown efficiency (Kennedy et al., 2004). See Table S4 for worm numbers and statistics. (C) *C. elegans* were grown from the L4 stage on the indicated RNAi bacteria – either in the presence of 50 µM Monorden or only its solvent (DMSO). The animals' survival was monitored. Changes in median lifespan of Monorden versus DMSO treatment are shown. Lifespan assays were performed using *eri-1(mg366)* mutant animals, to yield better knockdown efficiency (Kennedy et al., 2004). Significant lifespan changes ($p < 0.05$ (log-rank test)) are indicated (*). See Table S4 for drug concentrations, worm numbers, and statistics. (D) Eggs of wild type *C. elegans* were seeded onto plates treated with either 50 µM Monorden or solvent control (DMSO) and kept at 27ºC for 52 hours. Formation of Dauer larvae was scored based on their survival in 1% (w/v) SDS (results from biological quadruplicates, *: $p < 0.05$ (t-test), error bars indicate s.d.). See Table S4 for worm numbers and statistics.

Janssens, et al., Figure S5

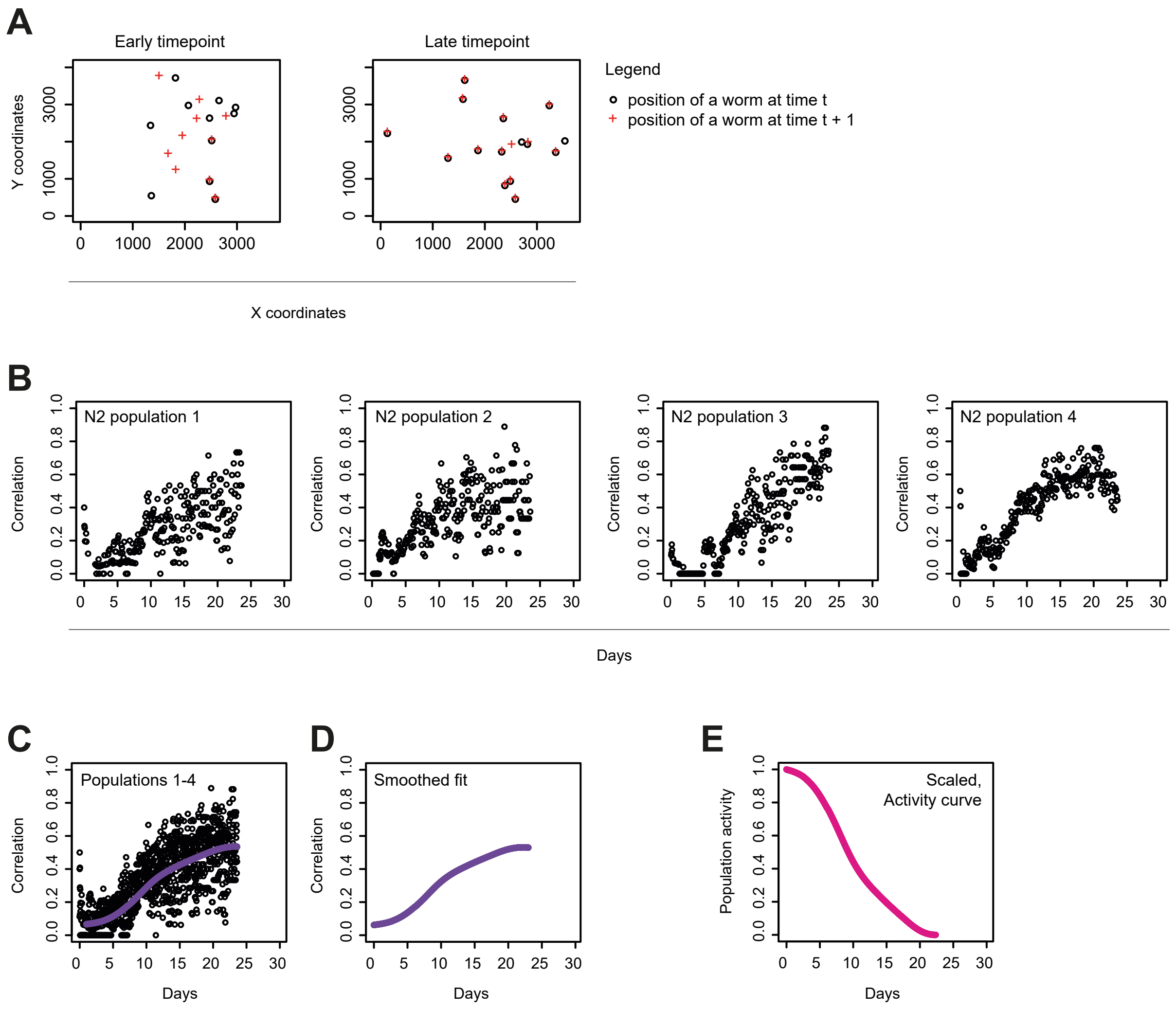

Figure S5. Population health span derived from the 'lifespan machine', related to Figure 4 and STAR Methods

This is an accompanying figure for steps described in the methods section. (A) Worm positions were assessed based on the position of worm objects identified in scanner images from the 'lifespan machine'. Left panel: Worm positions identified at an early time point (representing young animals). Abundant changes in worm locations can be seen between consecutive frames. Right panel: The same plot, but at a late time point (representing old animals), showing that worms now remain mostly in the same position. (B) Assessing correlations between subsequent time points as described in (A), shows trends towards increasing correlations in time, indicating less movement of the population. Four independent N2 worm populations are shown. (C) Merging all replicates and fitting a smoothed spline provides a general consensus of the population's trend. (D) The smoothed spline is considered independently and used to derive the graph shown in (E), by normalizing the starting point to 1 and end point to 0, generating a familiar 'curve' that describes the activity of worms at the population level.

Janssens, et al., Figure S6

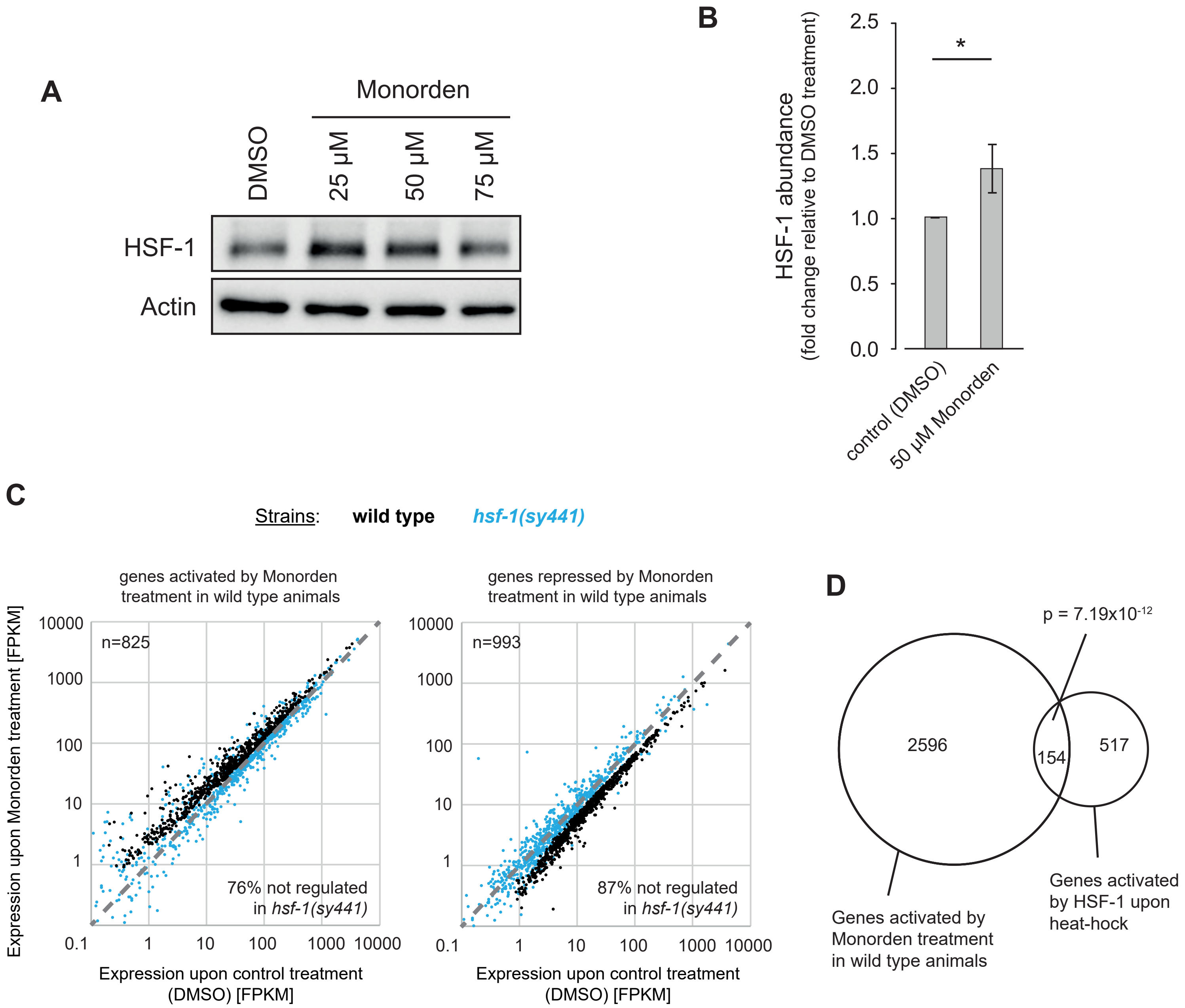

Figure S6. HSF-1 activation upon Monorden treatment, and analysis of Monorden-induced gene expression changes by mRNA-seq, both related to Figure 5

(A,B) Wild type *C. elegans* were grown from the L4 stage on different concentrations of Monorden or on solvent control (DMSO). At day 3 of adulthood, worms were harvested, whole worm lysates were prepared, and the lysates were subjected to SDS-PAGE and western blotting. (A) Representative western blot, showing changes in HSF-1 abundance upon treatment with Monorden. (B) Quantification of five independent experiments as shown in (A). HSF-1 abundance was normalized to actin abundance, and eventual fold changes relative to the control treatment with DMSO are shown (*: $p < 0.05$ (t-test), error bars indicate s.d.). (C) Wild type or *hsf-1(sy441)* mutant *C. elegans* were grown at 20°C and from the L4 stage exposed to either 50 µM Monorden or solvent control (DMSO). On day 3 of adulthood, animals were harvested and their transcriptomes determined by mRNA-seq. The scatter plots on the left show the genes significantly upregulated and the scatter plots on the right the genes significantly downregulated by Monorden treatment in wild type animals. It can be seen that many of these genes are not regulated in the *hsf-1(sy441)* background (The fraction of these genes is indicated.). (D) Wild type or *hsf-1(sy441)* mutant *C. elegans* were grown at 20°C until day 3 of adulthood, then shifted for 5 hours to 33°C, then harvested and their transcriptomes determined by mRNA-seq. Genes whose expression was impaired by the *hsf-1(sy441)* allele under these conditions were considered as 'Genes activated by HSF-1 upon heat-shock'. The Venn diagram shows that there is a signifi-cant overlap between these genes and the genes activated by Monorden treatment in wild type animals obtained under (A) ($p < 0.05$ (Fisher's exact test)).
