# Supplementary material for: Transcriptomics-Based Screening Identifies Pharmacological Inhibition of Hsp90 as a Means to Defer Aging
Source: Cell Rep. 2019 Apr 9;27(2):467–480.e6. doi: 10.1016/j.celrep.2019.03.044 (PMC6459000; doi:10.1016/j.celrep.2019.03.044)
Supplement: Table S5. Correlation between Geroprotector Prediction and Lifespan Extension in C. elegans, Related to Figure 3 [file mmc6.docx]

| **Drug** | **final_concentration_uM** | **lifespan_%_change** | **pvalue** | **p** | **adjusted_p** | **rank in predictions** |
| --- | --- | --- | --- | --- | --- | --- |
| tanespimycin | 25 | 54,16 | 0 | 7,00E-10 | 2,29E-07 | 3 |
| monorden | 50 | 25 | 0 | 4,29E-05 | 3,51E-03 | 8 |
| sirolimus | 50 | 16,14 | 0 | 8,55E-07 | 1,24E-04 | 6 |
| LY-294002 | 50 | 14,64 | 0 | 8,90E-09 | 2,33E-06 | 4 |
| valproic_acid | 50 | 13,24 | 0 | 1,01E-13 | 1,32E-10 | 1 |
| felbinac | 50 | 10,49 | 0,011 | 1,66E-02 | 1,89E-01 | 12 |
| trifluoperazine | 50 | 9,92 | 0,018 | 6,55E-06 | 7,15E-04 | 7 |
| estradiol | 50 | 9,77 | 0,367 | 9,48E-08 | 2,07E-05 | 5 |
| DL-PPMP | 50 | 5,99 | 0,057 | 6,99E-01 | 1,00E+00 | 16 |
| luteolin | 50 | 5,89 | 0,132 | 4,95E-02 | 3,37E-01 | 13 |
| trichostatin_A | 50 | 3,69 | 0,11 | 4,56E-11 | 2,98E-08 | 2 |
| wortmannin | 50 | 3,31 | 0,09 | 4,29E-05 | 3,51E-03 | 8 |
| dexverapamil | 50 | 2,18 | 0,448 | 8,83E-01 | 1,00E+00 | 16 |
| NU-1025 | 50 | 1,24 | 0,656 | 2,64E-01 | 8,54E-01 | 15 |
| haloperidol | 50 | 0,92 | 0,328 | 8,55E-07 | 1,24E-04 | 6 |
| isoflupredone | 50 | 0,37 | 0,147 | 4,95E-02 | 3,37E-01 | 13 |
| 5186223 | 50 | -0,97 | 0,593 | 6,99E-01 | 1,00E+00 | 16 |
| fulvestrant | 50 | -0,99 | 0,545 | 7,00E-10 | 2,29E-07 | 3 |
| apigenin | 50 | -1,9 | 0,927 | 1,25E-01 | 5,71E-01 | 14 |
| tretinoin | 50 | -2,18 | 0,706 | 4,29E-05 | 3,51E-03 | 8 |
| Prestwick-983 | 50 | -2,27 | 0,007 | 1,25E-01 | 5,71E-01 | 14 |
| genistein | 50 | -5,35 | 0,411 | 6,55E-06 | 7,15E-04 | 7 |
| fisetin | 50 | -5,94 | 0,083 | 1,00E+00 | 1,00E+00 | 16 |
| 1,5-isoquinolinediol | 50 | -7,34 | 0,026 | 1,00E+00 | 1,00E+00 | 16 |
| adiphenine | 50 | -8,9 | 0,019 | 1,66E-02 | 1,89E-01 | 12 |
| santonin | 50 | -9,88 | 0,003 | 6,55E-06 | 7,15E-04 | 7 |
